# Properties and Applications of Iron–Chalcogenide Superconductors

**DOI:** 10.3390/ma17133059

**Published:** 2024-06-21

**Authors:** Jianlong Zhao, Junsong Liao, Chiheng Dong, Dongliang Wang, Yanwei Ma

**Affiliations:** 1 Key Laboratory of Applied Superconductivity, Institute of Electrical Engineering, Chinese Academy of Sciences, Beijing 100190, China; 2 School of Electronic, Electrical and Communication Engineering, University of Chinese Academy of Sciences, Beijing 100049, China; 3 Institute of Electrical Engineering and Advanced Electromagnetic Drive Technology, Qilu Zhongke, Jinan 250013, China

**Keywords:** iron-based superconductors, critical current density, flux pinning, high-field applications

## Abstract

Iron–chalcogenide superconductors continue to captivate researchers due to their diverse crystalline structures and intriguing superconducting properties, positioning them as both a valuable platform for theoretical investigations and promising candidates for practical applications. This review begins with a comprehensive overview of the fabrication techniques employed for various iron–chalcogenide superconductors, accompanied by a summary of their phase diagrams. Subsequently, it delves into the upper critical field, anisotropy, and critical current density. Furthermore, it discusses the successful fabrication of meters-long coated conductors and explores their applications in superconducting radio-frequency cavities and coils. Finally, several prospective avenues for future research are proposed.

## 1. Introduction

**Figure 1:**
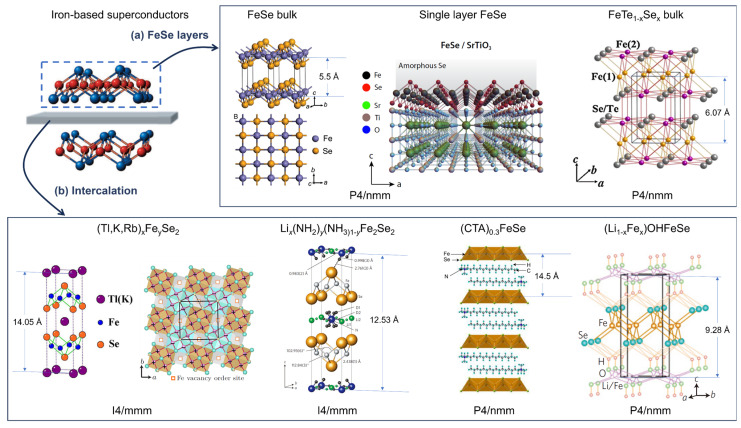
Crystal structures of iron-chalcogenide superconductors [2]. (**a**) FeSe-11 [6,7,8] and (**b**) intercalated FeSe superconductors [9,10,11,12,13].

Iron-based superconductors [1], discovered in 2008, are considered to be a new platform to study high-temperature superconducting mechanisms and potential candidates for practical applications [2,3,4]. From the crystalline structure point of view, iron-based superconductors can be divided into two major categories, iron–pnictides with the [FeAs]^−1^ layer and iron–chalcogenides with the electroneutral FeSe layer. The [FeAs]^−1^ layers in iron–pnictides are responsible for charge carrier transfer, which is alternated by the charge reservoir layers, e.g., the [ReO]^+1^, Ae^2+^, and A^+1^ layers (Re = rare earth elements, Ae = alkaline earth elements, A = alkaline elements) [5]. Consequently, there are varieties of iron–pnictides with multiple structures and abbreviations, such as 111, 122, 1111, 12,442, et al., as shown in Figure 1a. On the contrary, the iron–chalcogenides’ 11 system contains non-toxic elements and possesses the simplest crystal structure among iron-based superconductors, making them more attractive for theoretical studies and practical applications. Here, in this review, we start with the fabrication methods for iron–chalcogenide and discuss the superconducting transition temperatures related to different crystal structures and charge carrier densities. Then, the important factors for determining the applications, the upper critical field and the critical current density, are summarized for single crystals and films. At last, recent applications of FeTe_1−x_Se_x_-coated conductors are discussed.

## 2. Fabrications, Crystal Structures, and Superconducting Transition Temperature of Iron–Chalcogenide Superconductors

**Figure 2:**
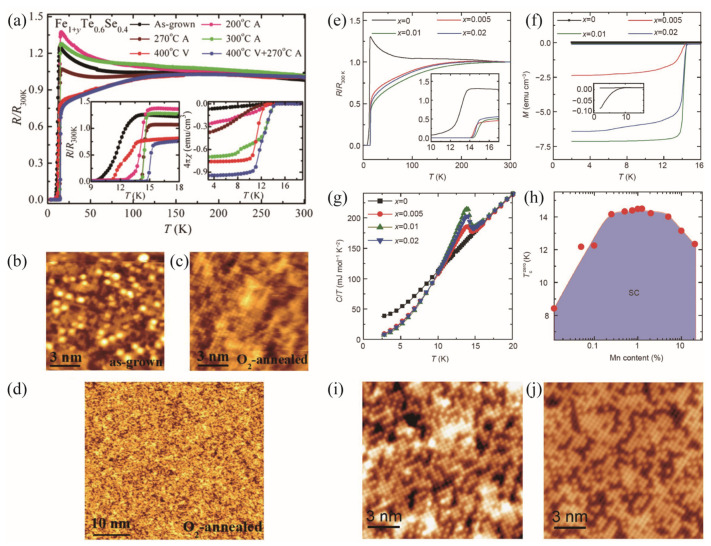
(**a**) R/R_300K_ for the FeTe_0.6_Se_0.4_ crystals before and after annealing under different atmospheres [19]. The left inset shows the data near T_c_, and the right inset plots the susceptibility χ(*T*). STM images for (**b**) as-grown and (**c**,**d**) O_2_-annealed Fe_1+*y*_Te_0.6_Se_0.4_ single crystal [16]. (**e**) Normalized resistance vs. temperature from 2 to 300 K for Fe_1−x_Mn*_x_*Te_0.55_Se_0.45_ (*x* = 0, 0.005, 0.01, and 0.02). The inset shows the resistance near the transition [15]. (**f**) The temperature-dependent susceptibility under a 10 Oe field for the samples with different Mn contents. (**g**) Temperature dependence of specific heat plotted as *C*/*T* vs. *T* for the Mn-doped samples. (**h**) Doping dependence of T_c_^zero^ of Fe_1−x_Mn*_x_*Te_0.55_Se_0.45_ (*x* = 0–0.2) single crystals. (**i**,**j**) STM topographic images of the cleaved single crystals with *x* = 0 (**left**) and 0.01 (**right**) at 250 mK.

The substitution of Se by Te or S induces novel superconductivity with higher superconducting transition temperatures, T_c_ [14,15]. However, there is a common characteristic in the 11 system, which is the excess Fe atoms (Fe(2) in Figure 1a) existing between the FeSe layers, as shown by the bright spots in the Scanning Tunneling Microscope (STM) images in Figure 2b,i [16]. They cause carrier localization and provide magnetic order deteriorating superconductivity in the Fe(1) plane [17], which masks the intrinsic superconductivity. It was soon found that annealing in air, O_2_, S, Se, Te, P, As, Sb, and I atmospheres can effectively remove the excess iron [18], as shown in Figure 2c,d. Correspondingly, the semiconductor-like normal state, namely, the resistivity (*ρ*), increases with cooling and changes to metallic-like behavior [19] with the increased annealing temperature, as shown in Figure 2a. Moreover, the T_c_ is enhanced from 9 K to 14 K, and the superconducting transition becomes sharper. The superconducting volume fraction of FeTe_0.6_Se_0.4_ approaches 100% after optimizing the annealing conditions. Gu et al. reported that through Mn element doping, the suppressed superconductivity in the pristine FeTe_0.55_Se_0.45_ single crystals can largely recover. As shown in the inset of Figure 2e, 1% Mn doping into the Fe site increases the T_c_^onset^ to 15 K. The quick saturation of susceptibility and the obvious jump in the temperature dependence of specific heat in Figure 2f,g suggest that bulk superconductivity emerges after the excess Fe atoms are removed, as evidenced by Figure 2j. They are summarized in a Mn-doping phase diagram, as shown in Figure 2h. Minor Mn doping of 0.5% has increased the T_c_ to a plateau value of 14.5 K, while further doping suppresses T_c_ and the jump of specific heat.

**Figure 3:**
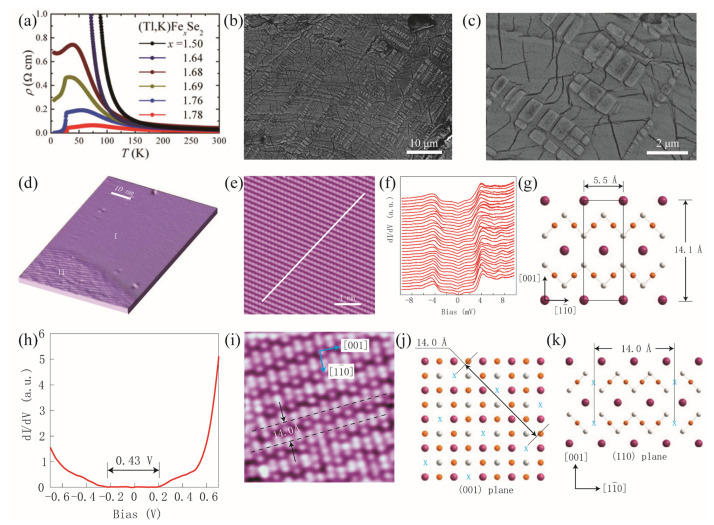
(**a**) The *ab*-plane resistivity *ρ*(*T*) as a function of temperature for (Tl,K)Fe*_x_*Se_2_ (*x* = 1.50, 1.64, 1.68, 1.69, 1.76, and 1.78) single crystals [9]. (**b**,**c**) Back-scattered electron images on the cleaved surface of the K_x_Fe_2-y_Se_2_ single crystals [20]. (**d**) STM topographic image of the K_x_Fe_2-y_Se_2_ film. Two distinct regions are marked by I and II [21]. (**e**) Atomic-resolution STM topography of region I. (**f**) Differential conductance spectrum along the white line in (**e**). (**g**) Atomic structure of the (110) plane. K and Se atoms are in the topmost layer. Fe atoms are in the second layer. (**h**) Differential conductance spectrum in region II. (**i**) Atomic-resolution STM topography of region II. (**j**) The structure of the $\sqrt{5}\times \sqrt{5}$ Fe vacancy pattern, as seen from (001) plane. (**k**) The positions of Fe vacancies are marked by crosses on the (110) plane.

The interlayer coupling of FeSe is dominated by the Van der Waals Force. The intercalation with metal atoms can be fulfilled through the traditional high-temperature synthesis routes and low-temperature ammonothermal techniques. The (Tl,K,Rb)_x_Fe_y_Se_2_ single crystals are usually fabricated through slow cooling from the melt at temperatures exceeding 900 °C. By increasing the Fe content, the normal state of (Tl,K)Fe_y_Se_2_ was tuned from an insulator to a semiconductor, accompanied by a superconducting transition at T_c_~31 K [9]. However, systematic investigation using a scanning electron microscope (SEM) and STM reveals a severe phase separation existing in this system. The filament KFe_2_Se_2_ superconducting phase (as shown by the rectangles in Figure 3b,c is sandwiched by the insulating K_2_Fe_4_Se_5_ phase with a Néel temperature of 560 K (the dark matrix) [20]. Li et al. found that the STM image in region I of Figure 3d exhibits a centered rectangular lattice structure with very few defects, as shown by Figure 3e. The Scanning Tunneling Spectroscopy (STS) reveals a double-gap structure, which is uniform along the white line in Figure 3e [21]. In region II, the STS indicates an energy gap of 0.43 eV across the Fermi level, suggesting an insulation phase. There are obvious Fe vacancies in the STM images, constructing a so-called $\sqrt{5}\times \sqrt{5}$ superlattice.

**Figure 4:**
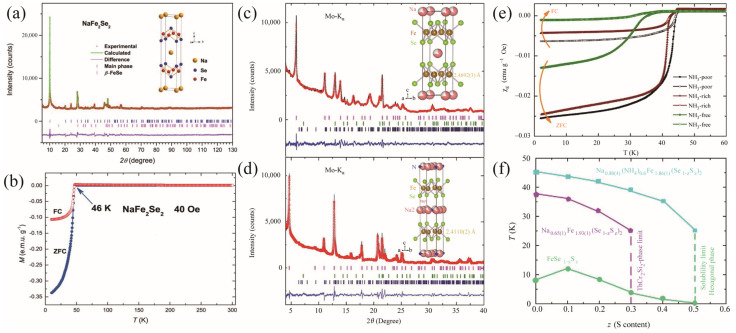
*NaFe_2_Se_2_ system*: (**a**) Powder XRD pattern and Rietveld refinement profile for nominal NaFe_2_Se_2_ at 297 K [23]. (**b**) Temperature dependence of susceptibility under ZFC and field-cooling (FC) procedures. The XRD patterns and Rietveld refinements for (**c**) the NH_3_-free Na_0.65(1)_Fe_1.93(1)_Se_2_ phase and (**d**) the NH_3_-poor Na_0.80(4)_(NH_3_)_0.6_Fe_1.86(1)_Se_2_ phase. (**e**) The magnetization curves of the samples with different NH_3_ contents. (**f**) T_c_ as a function of the S content in the FeSe_1−z_S_z_, Na_0.65(1)_Fe_1.93(1)_(Se_1−z_S_z_)_2_, and Na_0.80(4)_(NH_4_)_0.6_Fe_1.86(1)_(Se_1−z_S_z_)_2_ systems [24].

**Figure 5:**
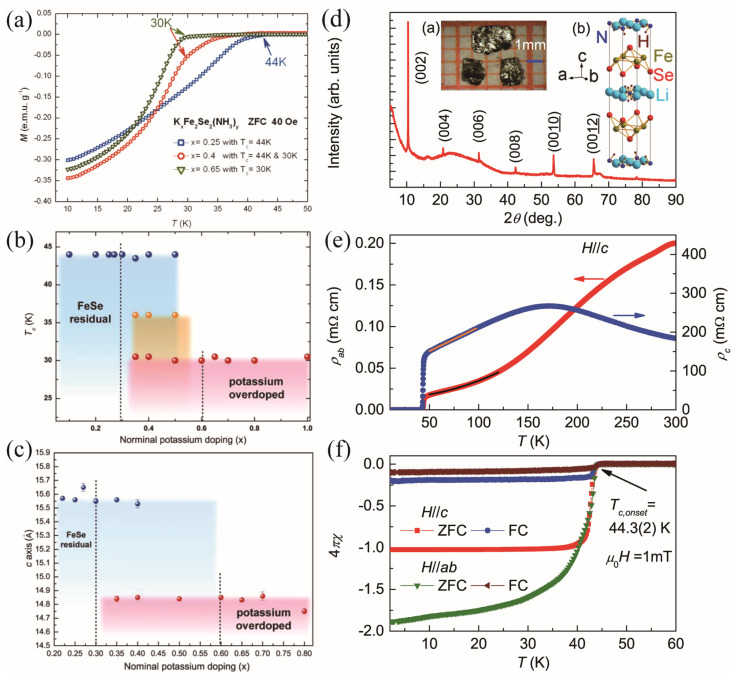
*K_x_Fe_2_Se_2_(NH_3_)_y_ system*. (**a**) M–T curves under a zero-field cooling (ZFC) procedure [24]. (**b**) T_c_ and (**c**) lattice constant *c* as a function of the nominal potassium content. *Li_x_(NH_3_)_y_Fe_2_Se_2_ single crystals*. (**d**) XRD pattern; the insets show the single crystals and the crystal structure. (**e**) Temperature dependence of in-plane resistivity *ρ_ab_*(*T*) and *c*-axial resistivity *ρ_c_*(*T*). (**f**) Temperature dependence of susceptibility under a 1 mT field for *H*//*c* and *H*//*ab* [25].

It is known that liquid ammonia dissolves alkali, alkaline earth, and some rare earth elements. Burrard-Lucas et al. first synthesized the Fe_1+δ_Se polycrystals using the solid-state-reaction method. The Fe_1+δ_Se and the Li metal were then placed in a Schlenk tube with a magnetic stirrer, which was attached to the ammonia cylinder. The tube was cooled to allow the ammonia to condense onto the reactants. The mixtures were then stirred at −78 °C for half an hour, after which the Schlenk tube was warmed up and the ammonia was evaporated. Finally, the Li*_x_*(NH_2_)*_y_*(NH_3)1-_*_y_*Fe_2_Se_2_ (*x*~0.6; *y*~0.2) with T_c_ = 43 K was obtained [11]. Ying et al., instead, directly poured the liquid ammonia into an autoclave filled with *β*-FeSe powders and alkali, alkaline earth, Yb, and Eu at liquid nitrogen temperature. The whole device was then kept at room temperature for 2–17 days [22]. Varieties of FeSe-based superconductors intercalated with Li, Na, Ba, Sr, Ca, Yb, and Eu were obtained, as proved by the Rietveld refinement shown in Figure 4a. The highest T_c_ is 45 K for NaFe_2_Se_2_. As shown in Figure 4b, the sharp superconducting transition and the large magnetization hysteresis loop indicate bulk superconductivity. By using the high-purity FeSe powders, they continued to intercalate K in FeSe via the ammonothermal route [23]. At least two superconducting phases were discovered, of which the T_c_ was controlled by the nominal content of K rather than the NH_3_. Discrete superconducting regions were revealed with abrupt changes of T_c_ with doping, as shown in Figure 5a,b. When *x* in K_x_Fe_2_Se_2_(NH_3_)_y_ is below 0.3, T_c_^onset^ is 44 K. When 0.3 < *x* < 0.6, a two-step transition was observed at T_c1_~44 K and T_c2_~30 K, indicating the phase separation in this doping region. There is only one superconducting phase with T_c_~30 K when *x* > 0.6. Moreover, there is no sign of antiferromagnetic (AFM) transition in the temperature-dependence of magnetization, indicating that the Fe-vacancy phase, such as the 245 phase, disappears in K_x_Fe_2_Se_2_(NH_3_)_y_. To further investigate the intrinsic superconducting properties, Sun et al. synthesize Li*_x_*(NH_3_)*_y_*Fe_2_Se_2_ single crystals through the low-temperature ammonothermal technique. The FeSe single crystals fabricated through the eutectic AlCl_3_ and NaCl (=0.52:0.48) flux method were used as the template. The as-synthesized thin plate-like Li*_x_*(NH_3_)*_y_*Fe_2_Se_2_ single crystals are shown in the inset of Figure 5d, with sharp (00*l*) diffraction peaks in the XRD patterns. The lattice parameters are *a* = 3.7704(6) Å and *c* = 16.973(7) Å. The T_c_^onset^ is determined to be 44.3 K. Surprisingly, the crystals exhibit a huge anisotropy in resistivity. The *ab*-plane resistivity *ρ*_ab_ exhibits a semiconductor behavior above 150 K, while the *c*-axis resistivity *ρ*_c_ presents a metallic behavior. Furthermore, the ratio *ρ*_c_/*ρ*_ab_ increases from 900 at 300 K to 8000 at 50 K, as shown in Figure 5e.

Guo et al. utilized a similar method to synthesize the NH_3_-free Na-intercalated FeSe_1-z_S_z_ superconductors. The difference is that the Taiatsu Glass TVS-N_2_ high-pressure vessel continued to be evacuated to ~10^−2^ Pa using a molecular pump after the reaction was finished [24]. The X-ray diffraction pattern shown in Figure 4c,d can be fitted well by Na_0.65_Fe_1.93_Se_2_ (91.3%), with minor impurities of FeSe (3.5%) and Fe_7_Se_8_ (5.2%). As depicted in Figure 4e, the NH_3_-free phase has a T_c_ of 37 K, while the NH_3_-poor intercalates show a T_c_ of 45 K. Figure 4f summarizes the S-doping-dependence of T_c_ for the FeSe_1-z_S_z_ and two intercalates. With S doping, the T_c_ decreases quicker in the NH_3_-free phase than in the NH_3_-poor phase, indicating that S doping effect is weaker in the NH_3_-poor phase. 

Except for the low-temperature ammonothermal method, Hatakeda et al. dissolved lithium and sodium metal in 2-phenethylamine (2-PEA) and reacted them with FeSe powders at 45 °C for 7 and 28 days, respectively [26]. The *c*-axis length is 19.04(6) Å and 18.0(1) Å for the Li- and Na-cases, respectively, which is the largest among the iron–chalcogenide superconductors. T_c_ is 39 K for the Li-case, while a two-step transition is observed in the Na-case. The thermalgravimetric measurements show a 27% mass loss below 300 °C, which is ascribed to the deintercalation or desorption of 2-PEA, and a nearly 60% mass loss above 700 °C. This implies a high instability of the intercalated FeSe at high temperatures.

**Figure 6:**
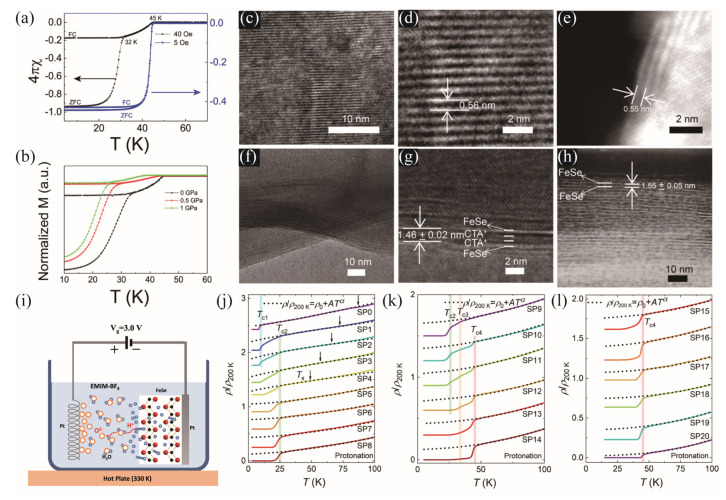
(**a**) Temperature dependence of susceptibility of (CTA)_0.3_FeSe under an external field of 5 and 40 Oe. (**b**) The normalized susceptibility of (CTA)_0.3_FeSe at different external pressures. (**c**,**d**) The high-resolution TEM (HRTEM) images and (**e**) the STEM-HAADF (high-angle annular dark field) image of the pristine FeSe crystals. (**f**,**g**) The HRTEM images and (**h**) the STEM-HAADF image of the (CTA)_0.3_FeSe crystals [12]. The brighter layer is the FeSe layer and the darker layer is the CTA^+^ organic layer. (**i**) Schematic of the experimental setup used for the protonation of FeSe. (**j**–**l**) Temperature dependence of the normalized resistivity, *ρ*(*T*)/*ρ*_200K_, for H*_x_*-FeSe single crystals during the protonation process [27].

Shi et al. performed an electrochemical intercalation method to successfully synthesize a cetyltrimethyl ammonium (CTA^+^)-intercalated FeSe-based superconductor (CTA)_0.3_FeSe with T_c_ = 45 K [12]. The crystal structure is exhibited in Figure 1b. The FeSe single crystals were used as the starting materials and fixed on an In wire as the positive electrode. The electrolyte is N-methyl-2-pyrrolidone added by hexadecyl trimethyl ammonium bromide (CTAB). The Transmission Electron Microscope (TEM) indicated that the distance between the FeSe layers increased from 0.56 nm for the pristine FeSe to 1.45 nm for the CTA^+^ intercalated FeSe. The CTA^+^ organic layer can be clearly seen, as shown by the darker layer in Figure 6g. Figure 6a shows the temperature dependence of susceptibility measured under the fields of 5 Oe and 40 Oe. At 5 Oe, the T_c_ is determined to be 45 K, and the small distance between the FC and ZFC branches implies weak flux-pinning properties, confirming the high degree of homogeneity of the intercalated sample. The T_c_ also exhibits a negative pressure effect up to 1 GPa, which is consistent with other pressure experiments on the intercalated FeSe systems under low pressure. Meng et al. cleaved the FeSe single crystals obtained through the chemical vapor transport (CVT) method into thin flakes with a thickness of ~15 μm [27]. Several crystals were attached to the negative electrode using silver paint. The ionic liquid 1-ethyl-3-methylimidazolium tetrafluoroborate (EMIM-BF_4_) was used as the electrolyte, as schematized in Figure 6i. By using the optimized voltage 3 V and a temperature of 330 K provided by a thermostatic hot plate, the intercalated FeSe samples with different H^+^ content were obtained after different protonation times. As depicted in Figure 6j–l, the nematic transition at T_s_~89 K marked by the black arrows in (j) was gradually suppressed after protonation and totally disappeared after 5 days. Meanwhile, the T_c_ is enhanced from 10 K for the pristine FeSe single crystals to T_c1_ = 25 K at 5 days of protonation. In the intermediate protonation time, there is a coexistence of three superconducting transitions with T_c2_ = 25 K, T_c3_ = 34 K, and T_c4_ = 44 K. After 15 days, only the T_c4_ = 44 K phase survived. Moreover, the magnetization measurements prove the 100% volume fraction of superconductivity for all of the samples at 2 K. They also proved that this effect was reversible after deprotonation, as the T_c4_ phase gradually transformed to the T_c1_ phase, accompanied by the reappearance of the nematic order.

**Figure 7:**
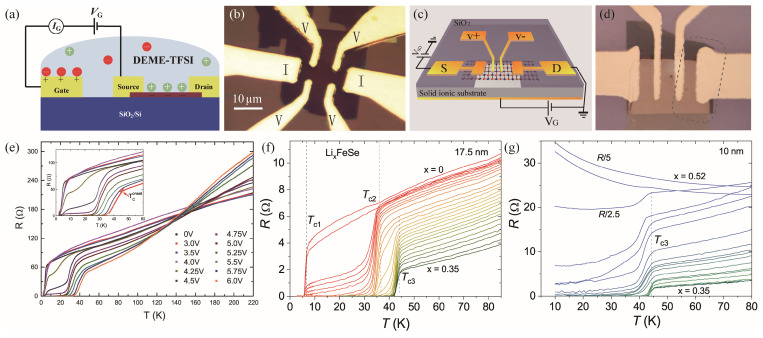
(**a**) A schematic illustration of the FeSe thin flake EDLT device in Ref. [28]. The ionic liquid DEME-TFSI serves as the dielectric, covering the sample and gate electrodes. (**b**) The optical image of the EDLT device. (**c**) Schematic structure and (**d**) optical image of the solid ionic gating device in Ref. [29]. From the bottom to the top: silver back gate layer, Li/Na-ion substrate, FeSe thin flake, 100 nm SiO_2_, and four electrodes. The gate voltage *V*_G_ is applied at low temperatures (<155 K), where all of the ions in the substrate are frozen in place. (**e**) Temperature dependence of the resistance for a FeSe thin flake at different *V_g_*. (**f**) Resistance of the Li-intercalated FeSe flake (17.5 nm) as a function of temperature. (**g**) Resistance of another 10 nm FeSe flake in the overdoped regime [29].

**Figure 8:**
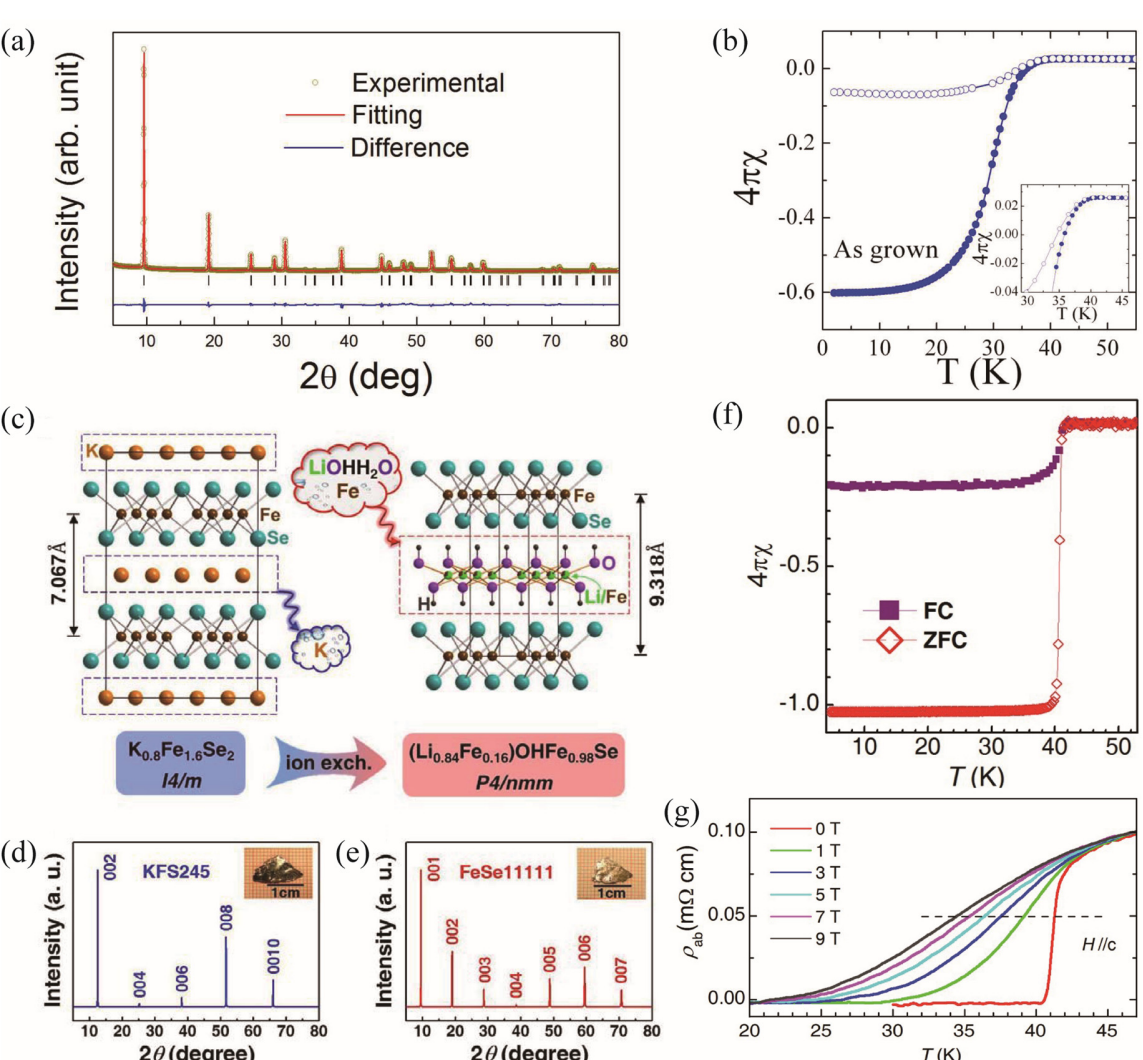
(**a**) XRD and Rietveld refinements of LiFeO_2_Fe_2_Se_2_ [30]. (**b**) Temperature dependence of susceptibility for the as-grown (Li_0.8_Fe_0.2_)OHFeSe [13]. (**c**) Hydrothermal ion-exchange process [32]. XRD of the (**d**) K_2_Fe_4_Se_5_ and (**e**) (Li_0.84_Fe_0.16_)OHFe_0.98_Se single crystals. (**f**) Susceptibility and (**g**) magnetoresistivity of the (Li_0.84_Fe_0.16_)OHFe_0.98_Se single crystals.

Lei et al. applied the electric-double-layer transistor (EDLT) using ionic liquids as the gate dielectric to the single crystalline FeSe flakes with typical thicknesses of ~10 nm [28], as shown in Figure 7a,b. The ionic liquid DEME-TFSI was used as the dielectric, and the cations and anions were DEME^+^ and TFSI^-^, respectively. After introducing carriers to the FeSe flakes at 220 K with the application of gate voltages, the sheet resistance was measured, as depicted in Figure 7e. After increasing the gate voltage V_g_, the pristine T_c_ = 5.2 K was enhanced to 7.5 K at V_g_ = 4.0 V. Further increase of V_g_ induces a two-step transition. Above V_g_ > 5.0 V, there is only one superconducting phase with T_c_ above 40 K. The highest T_c_~48.2 K is realized after applying V_g_ = 6 V. Similar results were observed in the Li/Na intercalated FeSe superconductors by using the ionic gating technique [29]. The lithium-based Li_1+x+y_A_x_(Ti,Ge)_2−x_Si_y_P_3−y_O_12_ and the sodium-based Na_3.4_Zr_1.8_Mg_0.2_Si_2_PO_12_ were adopted as the substrates, as shown in Figure 7c,d. The 5–38 nm thick FeSe flake was sandwiched between the solid ionic substrate and a 100 nm SiO_2_ layer. The SiO_2_ layer is crucial to insulate the electrodes from the substrate to avoid metal accumulation and accurate determination of the content of the intercalated metals. It can be observed that with intercalation of Li cations, the T_c1_ = 8 K from the FeSe matrix gradually increases to T_c2_ = 36 K and finally enters into the phase with T_c3_ = 44 K, accompanied by decreased normal state resistivity. However, the normal state resistance gradually increases upon Li doping, and, finally, the T_c3_ = 44 K phase disappears and the sample transforms into an insulator, as shown in Figure 7g. Similar intercalation behavior was found in Na_x_FeSe. The common discrete superconducting phase in these systems was found to be robust against S substitution but vulnerable to the Fe site substitution by Cu, implying the importance of the intact Fe plane to the high-temperature superconductivity.

(Li_1−x_Fe_x_)OHFeSe was first prepared through the hydrothermal reaction method. Selenourea, Fe powders, LiOH·H_2_O, and de-ionized water were put into an autoclave and heated at 160 °C for 3–10 days [30]. The crystal structure was determined through XRD and Rietveld refinements (Figure 8a). As depicted in Figure 1b, the anti-PbO-type FeSe layers are alternated with the anti-PbO-type LiFeO_2_. The crystal lattice parameters are determined to be *a* = 3.7926(1) Å, *c* = 9.2845(1) Å. The susceptibility shows a superconducting transition at 40 K. Sun et al. systematically studied the superconducting properties of (Li_1−*x*_Fe*_x_*)OHFe_1-*y*_Se with different iron vacancy concentrations [31]. It was found that the synthesized compounds through hydrothermal synthesis lead to *x*~0.2, but with a variable *y*. Interestingly, superconductivity emerges when Fe vacancy (*y* < 0.05) is decreased. The lithiation process reduces *y* to zero by replacing some Fe ions in the reservoir layer by Li, while the replaced Fe fills into the vacancy in the conducting layer. As a result, a large superconducting volume was reached with the highest T_c_. Due to the powder form, the resistivity measurements on the pressed bulk show a low T_c_^zero^. Dong et al. successfully synthesized single crystalline (Li_0.84_Fe_0.16_)OHFe_0.98_Se via the hydrothermal ion-exchange technique based on the K_0.8_Fe_1.6_Se_2_ (245 insulating phase) single crystals [32]. During the hydrothermal reaction process, the K ions in the 245 single crystals were completely released into the solution, and (Li/Fe)OH layers constructed by ions from the solution were squashed into the matrix, which connects with the adjacent edge-sharing FeSe tetrahedra via weak hydrogen bonding, as schematized in Figure 8c. Despite the sharp superconducting transition at 42 K in the susceptibility data, clear resistivity evidence was also observed, as shown in Figure 8g.

**Figure 9:**
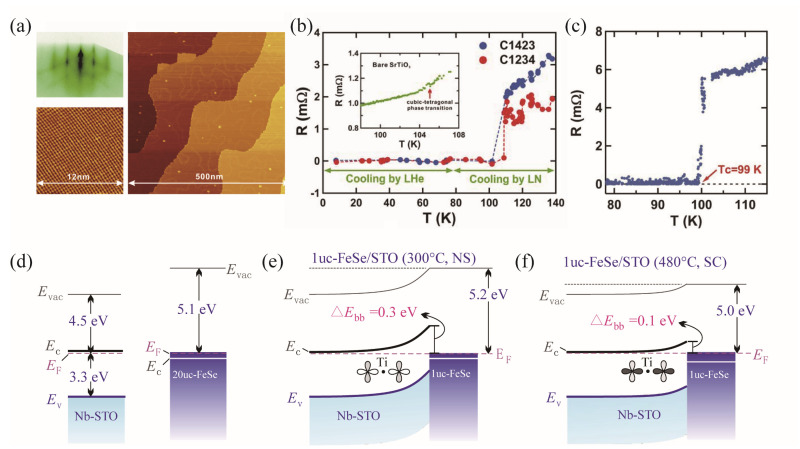
(**a**) Top left: RHEED pattern after the growth of single-layer FeSe. Bottom left: atomic-scale STM image of the film. Right: large-scale STM image. (**b**) R–T curves derived from the I–V curve measured at fixed temperatures [36]. (**c**) Similar R–T curve but measured by sweeping the temperature. (**d**) Energy bands of Nb-doped STO and 20 uc-FeSe separately. (**e**,**f**) Energy band profile across the FeSe/STO heterostructure at the non-superconducting and superconducting stages, respectively [35].

The highest T_c_ in iron–chalcogenide superconductors was obtained in the single-layer FeSe film prepared through the Molecular Beam Epitaxy (MBE) method [33,34]. The FeSe single-layer film was grown on the TiO_2_-terminated and Nb-doped SrTiO_3_ (STO-001) substrate at 450 °C by co-evaporating Fe and Se from Knudsen cells with a flux ratio of 1:10. RHEED was used to monitor the growth process, as shown in Figure 9a. Figure 9b shows the temperature dependence of resistance derived from the I–V curves measured at fixed temperatures. The T_c_ is determined to be 109 K. Another R–T curve with T_c_ = 99 K was obtained through measurements conducted while sweeping the temperature, as shown in Figure 9c. As a result, it can be claimed that this is the second high-temperature superconductor with T_c_ surpassing the liquid nitrogen temperature, indicating great significance not only for the superconducting mechanism but also for potential applications in the liquid nitrogen temperature region. Xue’s group continues to investigate the mechanism [35] of high-temperature superconductivity [35]. They suggest that band bending of STO occurs due to the larger work function of FeSe compared to STO, which contributes to the charge transfer to the FeSe side, as shown in Figure 9d. Moreover, annealing induces stoichiometry of FeSe and shifts the Fermi level upward, thus increasing the electron density (Figure 9e,f). Consequently, the high-temperature superconductivity emerges in the superconducting/dielectric heterostructure, where the dielectric layer enhances the charge density of FeSe and provides the electron–phonon coupling with its intrinsic high-energy phonon mode.

## 3. Phase Diagram of Iron–Chalcogenide Superconductors

**Figure 10:**
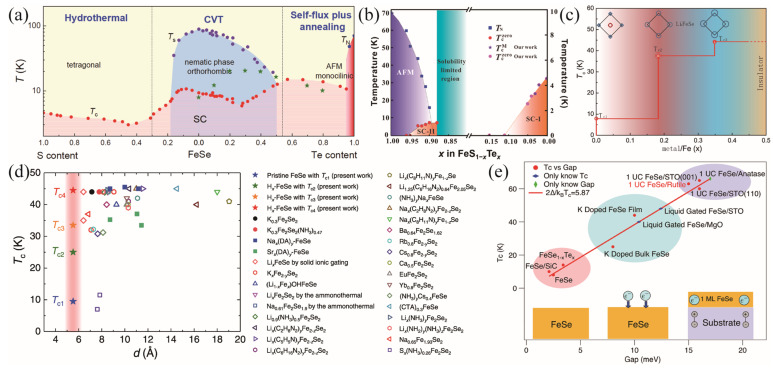
Phase diagrams of iron–chalcogenide superconductors. (**a**) The entire phase diagram of FeSe_1−*x*_Te*_x_* and FeSe_1−*x*_S*_x_* single crystals synthesized through the optimal methods, hydrothermal for FeSe_1−*x*_S*_x_* (0.29 ≤ *x* ≤ 1), CVT for FeSe_1−*x*_S*_x_* (0 ≤ *x* ≤ 0.29) and FeSe_1−*x*_Te*_x_* (0.29 ≤ *x* ≤ 1), and self-flux plus annealing for FeSe_1−*x*_Te*_x_* (0.55 ≤ *x* ≤ 1) [37]. The data from the FeSe_1−x_Te_x_ film are also included, as marked by the green pentagon [41]. (**b**) The doping phase diagram of FeS_1−*x*_Te*_x_* single crystals [37]. (**c**) Phase diagram of FeSe-derived superconductors [29]. The red lines denote the discrete SC phases of metal-intercalated FeSe superconductors, as represented by Li*_x_*FeSe. The different Fermi surface topologies of the pristine and doped FeSe are superimposed. The yellow area on the right represents the insulating phase in the heavily overdoped region. (**d**) T_c_ vs. interlayer distance *d* in the intercalated FeSe superconductors [27]. (**e**) T_c_ as a function of the maximum superconducting gap of various FeSe-based superconductors [47].

The phase diagrams of iron–chalcogenide superconductors are summarized in Figure 10. The left side of Figure 10a shows the FeSe_1−x_S*_x_* single crystals fabricated through the hydrothermal route (0.29 ≤ x ≤ 1) and the CVT method (0 ≤ *x* ≤ 0.29) [37]. In the nematic phase below T_s_, the normal state resistivity presents a linear temperature dependence, namely, the non-Fermi liquid behavior [38]. With S doping, the nematic order is gradually suppressed, and the Fermi liquid behavior is recovered. A small superconducting dome appears within the nematic phase, with the highest T_c_~9 K. It reaches a minimum value at *x*~0.45 and increases again to the FeS end. The right side of Figure 10a shows the Te doping dependence of T_c_ and T_N_. The post-annealing process applied to the high Te content of FeSe_1−x_Te_x_ single crystals prepared through the self-flux method proves the coexistence of long-range AFM order and superconductivity. However, high Se side crystals must be synthesized through the CVT method to avoid phase separation [39,40]. The T_c_ of FeSe_1−x_Te*_x_* presents a minimum around *x*~0.2 due to the sample disorder. The nematic order is also gradually suppressed by Te doping and disappears at *x*~0.5, where the T_c_ reaches the maximum value of 15 K. Zhuang et al. also obtained a full doping phase diagram of FeSe_1−x_Te_x_ by synthesizing the film on the CaF_2_ (100) substrate using the Pulsed Laser Deposition (PLD) method [41]. As marked by the green pentagon in Figure 10a, the T_c_ shows a maximum value of 20 K within 0.6 ≤ *x* ≤ 0.8, where phase separation should be apparent. High transition temperatures with T_c_~24 K were obtained in FeTe_0.8_Se_0.2_ film [42,43]. The difference between the film and the CVT single crystals may originate from the strain effect induced by the substrate. Figure 10b shows the Te doping phase diagram of FeS_1−x_Te_x_. Similarly to the doped FeSe, S doping in FeTe gradually suppresses the AFM order, while superconductivity appears when *x* < 0.95 [44]. There is a region of coexistence of AFM and superconductivity. The maximum T_c_ is 9 K. However, the solubility limit of S in FeTe prevents further investigation into this system. Zhao et al. prepared the FeS_1−x_Te_x_ single crystals with 0 ≤ *x* ≤ 0.15 via the hydrothermal technique [45]. The T_c_ = 4.5 K in FeS [46] was quickly suppressed after Te doping due to the scattering from the impurities, and the superconductivity was totally suppressed when *x* > 0.1.

Ying et al. summarized a common electron doping phase diagram of the intercalated FeSe superconductors, as shown in Figure 10c. An obvious discreet superconducting phase diagram can be seen [27,29]. The corresponding Fermi surface topologies are also incorporated at the top. For the pristine FeSe system, the hole pockets are at the center of the Brillouin zone (Γ point), and the electron pockets are at the corner (M points). Upon electron doping to a critical level, the Lifshitz transition occurs and leads to an abrupt appearance of the T_c2_ phase. Further increasing the doping level induces the enlarged electron pocket and the emergence of the T_c3_ phase. Figure 10d depicts T_c_ as a function of the interlayer distance, *d*, for all of the FeSe-based superconductors published so far. It seems that the T_c_ is enhanced with the interlayer distance when *d* < 9 Å, but it saturates with the higher *d*. For the protonated FeSe sample, the T_c_ is enhanced by increasing the electron density, rather than the interlayer distance, as proved by the enhancement from T_c1_ to T_c4_ with the same interlayer distance. Rebec et al. explain the phase diagram from the viewpoint of the superconducting energy gap, Δ [47]. As schematized in Figure 10e, there is a linear T_c_ dependence on the gap 2Δ/k_B_T_c_ = 5.87, which demarcates the phase diagram into three regions. For region I, the FeSe/Te bulks exhibit a Δ < 5 meV, and the T_c_ is less than 20 K. In the second region, a gap of Δ~10 meV is opened at a low temperature, and the T_c_ of the intercalated or electron-doped FeSe is enhanced to 31–48 K [48]. The 1-unic cell FeSe on the STO substrate belongs to the third group, where interfacial electron–phonon interaction plays an important part in high-temperature superconductivity.

## 4. Upper Critical Field, Anisotropy, and Critical Current Density of Practical Iron–Chalcogenide Superconductors

**Figure 11:**
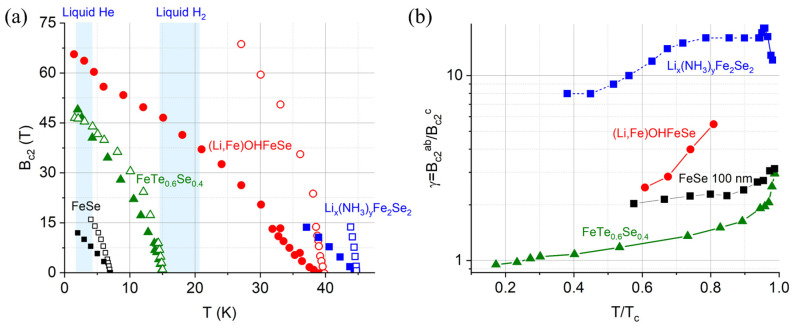
(**a**) Upper critical field, B_c2_, of iron–chalcogenide superconductors [23,49,50,51]. The empty and solid symbols denote the field parallel to the *ab*-plane and the *c*-axis, respectively. (**b**) Anisotropy parameter, γ = B_c2_^ab^/B_c2_^c^, as a function of normalized temperature T/T_c_.

Two crucial parameters related to practical applications are the upper critical field, above which Cooper pairs are depaired at high magnetic fields, and the anisotropy parameter, γ = B_c2_^ab^/B_c2_^c^ = (m_c_/m_ab_)^1/2^ = λ_c_/λ_ab_ = ξ_c_/ξ_ab_, where m, λ, and ξ are the effective mass of electrons, the penetration depth, and the coherence length, respectively. Figure 11a summarizes the temperature dependence of the upper critical field, B_c2_. One similarity is that the B_c2_ with the field parallel to the *ab*-plane of the crystal structure is larger than the field parallel to the *c*-axis. This is caused by the effective mass anisotropy of electrons. For the FeSe (100 nm thick flake), the B_c2_ is 16 T at 4 K when the field is parallel to the *ab*-plane. [49]. The B_c2_ with the field parallel to the *c*-axis is smaller, achieving a B_c2_ = 12 T at 2 K. Te doping in the Se site not only enhances the T_c_ but also increases the B_c2_ to 50 T at 2 K. Moreover, the B_c2_^ab^ is closer to the B_c2_^c^ below T_c_ than FeSe, and there is a crossover between them at 3 K. For the intercalated FeSe superconductors, the enhanced T_c_ makes the application in liquid hydrogen possible. For the (Li,Fe)OHFeSe single crystals, the B_c2_^c^ is increased to 45 T and 65 T at the liquid hydrogen and liquid helium temperature regions, respectively [50]. The B_c2_^ab^, on the other hand, is 68 T at 27 K, indicating good application potential. The data of Li_x_(NH_3_)_y_Fe_2_Se_2_ are limited below 14 T, but there is an apparent large gap between the two directions [25].

The anisotropy parameters of the above-mentioned superconductors are depicted as a function of normalized temperature, *t* = T/T_c_, in Figure 11b. For the FeTe_0.6_Se_0.4_ single crystals, the γ increases from values smaller than 1 (reversed anisotropy with B_c2_^ab^ < B_c2_^c^) below 0.25 T_c_ to 1 < γ < 2, and finally above γ = 2 near T_c_. The FeSe flakes with infinite conducting layers exhibit more two-dimensional characteristics and exhibit a γ > 2 in the whole studying range. The nearly isotropic behavior of B_c2_ is beneficial to the magnet design. For the (Li,Fe)OHFeSe with larger interlayer space, the γ increases from 2.5 to 5.5 when *t* = T/T_c_ moves from 0.6 to 0.8. The largest γ is observed in Li_x_(NH_3_)_y_Fe_2_Se_2_, ranging from 8 to 18 above *t* = 0.38 [25]. The extremely large electronic anisotropy in both (Li,Fe)OH and Li-NH_3_ layers of intercalated FeSe compounds is suggested to result from the structure’s characteristics.

In the next section, we will discuss the most important parameter, the critical current density of iron–chalcogenide superconductors, reported so far. Although the *J*_c_ has already been studied in the (Tl,K,Rb)_x_Fe_y_Se_2_ system [52,53,54], the discussion will not be included here because of the severe phase separation and the small portion of the superconducting phase, which leads to a small *J*_c_~10^4^ A/cm^2^ calculated through the Bean model. Instead, the (Li,Fe)OHFeSe system with an intact Fe plane and 100% volume fraction of superconductivity will be discussed.

Generally, the upper limit of critical current density is determined through the depairing critical current density, which is described by

$$J_{d}\left(0\;K\right)=\frac{\phi_{0}}{3\sqrt{3}\mu_{0}\lambda_{ab}^{2}\left(0\;K\right)\xi_{ab}(0\;K)}$$

where Φ_0_ is the flux quantum. The penetration depth and coherence length of three typical iron–chalcogenide superconductors are listed in Table 1. The ξ of FeSe is two to three times those of FeTe_0.5_Se_0.5_ and (Li,Fe)OHFeSe, leading to a small *J*_d_ of 11 MA/cm^2^. Comparatively, the *J*_d_ of the FeTe_1−x_Se_x_ and (Li,Fe)OHFeSe system achieves 36 and 57 MA/cm^2^ at 0 K.

However, the actual *J*_c_ a superconductor can carry is mainly controlled by the flux pinning efficiency of the pinning landscape, in which the dimensionality also determines the *J*_c_ anisotropy. As summarized in Table 1, the pristine FeSe single crystals only achieve a small *J*_c_ of 0.043 MA/cm^2^. After the irradiation of 2.4 GeV U ions, the *J*_c_ approaches the same level of the pristine FeTe_0.61_Se_0.39_ and (Li,Fe)OHFeSe single crystals [61]. Meanwhile, the irradiated FeTe_0.61_Se_0.39_ single crystals by 200 MeV Au achieve 0.5 MA/cm^2^ at 0 T and 2 K. The FeTe_1−x_Se_x_ film and coated conductor have a higher *J*_c_ above 1 MA/cm^2^ because of the more abundant crystal defects incorporated after the PLD fabrication process.

**Figure 12:**
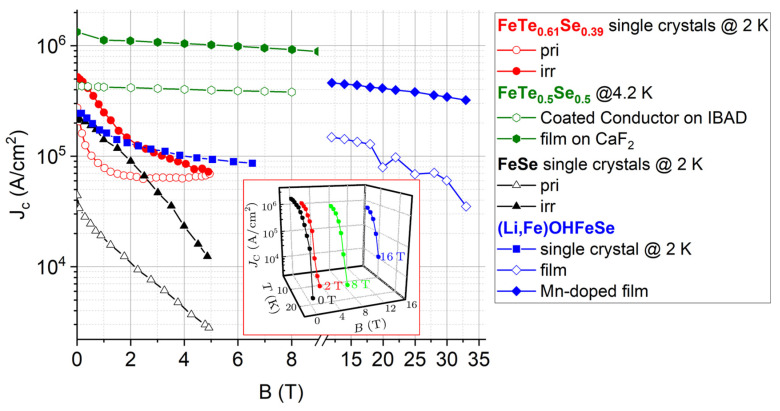
Field dependence of *J*_c_ for iron–chalcogenide superconductors [58,61,62,63,64,69]. The inset shows *J*_c_ of the single-layer FeSe [34].

Figure 12 summarizes the critical current density of the state-of-the-art iron–chalcogenide superconductors. Generally speaking, the iron–chalcogenide single crystals have much fewer crystalline defects than the film, leading to a weak flux pinning force, strong field dependence, and low *J*_c_ at high fields. The FeSe single crystals show a *J*_c_(2 K) = 4 × 10^4^ A/cm^2^ at 0 T, which decreases quickly with the field. The FeTe_1−x_Se_x_ single crystals, on the contrary, exhibit a robust field dependence above 2 T. Irradiation is an effective way to introduce artificial pinning centers into the ‘11’ system, leading to a pronounced enhancement of its current carrying ability. The *J*_c_ of FeSe at high fields has been enhanced four times. For the irradiated FeTe_1−x_Se_x_ single crystals, the Au irradiation has increased the *J*_c_ below 5 T, above which the *J*_c_ before and after irradiation intersect with each other. The (Li,Fe)OHFeSe single crystals have a higher *J*_c_ than the FeTe_1−x_Se_x_ single crystals in the testing range due to the intrinsic pinning, which will be discussed below [63]. Comparatively, the iron–chalcogenides in the film form have higher *J*_c_ because of the strong flux pinning centers constructed during the film growth [42,64,66,67,68]. The FeTe_1−x_Se_x_ film achieves a high *J*_c_(0 T) = 1.36 MA/cm^2^ and exhibits a rather robust field dependence [69]. Even at 9 T, the *J*_c_ remains 0.97 MA/cm^2^. The (Li,Fe)OHFeSe film grown through the hydrothermal method shows a *J*_c_ below 10^5^ A/cm^2^ above 20 T [58]. However, the extrapolation to the low field region seems higher than the (Li,Fe)OHFeSe single crystals. Moreover, the Mn doping considerably enhances the high field *J*_c_ by nearly 10 times at 33 T, which is ascribed to the extra pinning centers introduced by Mn doping [58]. A similar Mn doping effect on *J*_c_ is observed in the FeTe_1−x_Se_x_ [15] and K_x_Fe_2−y_Se_2_ [53] systems, but the former is due to the extinguished excess Fe irons. Interestingly, the single-layer FeSe film has a *J*_c_ above 1 MA/cm^2^ at self-field. Such a high *J*_c_ in the one-unit cell FeSe slowly declines with fields, as shown in the inset.

**Figure 13:**
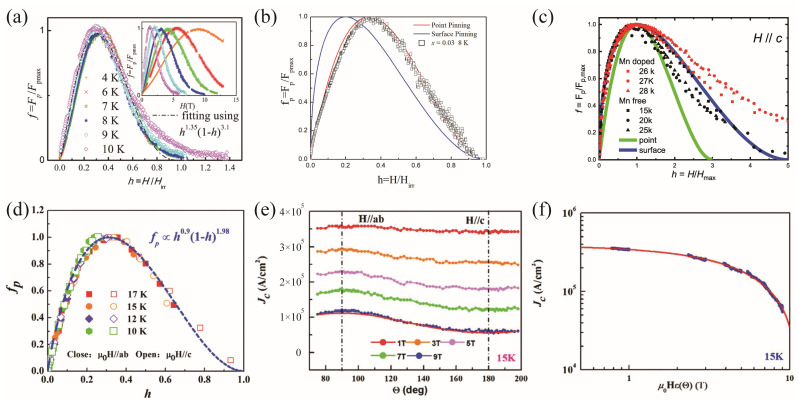
(**a**–**d**) Normalized flux pinning force *f* = *F*_p_/*F*_p,max_ as a function of reduced field *h* = *H*/*H*_max_ for (**a**) the Fe_1.04_Te_0.6_Se_0.4_ single crystal [70], (**b**) the 3at. % Co-doped FeSe_0.5_Te_0.5_ single crystal [71], (**c**) the Mn-doped and Mn-free (Li,Fe)OHFeSe films [58], and (**d**) the FeSe_0.5_Te_0.5_ film. (**e**) Angle dependence of *J*_c_ and (**f**) *J*_c_ vs. effective field, H_eff_ of the FeSe_0.5_Te_0.5_ film [67].

The flux pinning mechanism is important to unravel the factors governing the critical current density. Figure 13 presents the flux pinning properties of the FeTe_1−x_Se_x_ and (Li,Fe)OHFeSe single crystals and films. The flux pinning type of superconductors is usually studied through the Dew–Hughes model or the angle dependence of *J*_c_. The flux pinning mechanism can be obtained according to the peak of the *f*_p_-*h* curve, where *f*_p_ is the normalized flux pinning force *f*_p_ = F_p_/F_p_^max^ and *h* is the reduced magnetic field *h* = H/H_irr_. The Fe_1.04_Te_0.6_Se_0.4_ single crystals have a *h*_peak_ = 0.3, indicating mixed pinning from the point and surface defects [70]. After 3at. % Co doping into the Fe sites, the enhanced *J*_c_ is still dominated by the point defects [71]. The FeTe_0.5_Se_0.5_ film deposited on the CaF_2_ film also shows a peak at *h*_peak_ = 0.33, suggesting a point pinning mechanism, as shown in Figure 13d. The angular *J*_c_ at 15 K in Figure 13e has a peak at the fields parallel to the *ab*-plane [67] due to the intrinsic pinning. As evidenced in Figure 13f, the *J*_c_(Θ, H) curves collapse into one curve when it is plotted against the effective field, H_eff_ = Hε(Θ), where ε(Θ) = (cos^2^Θ + γ^−2^sin^2^Θ)^1/2^. The fitted anisotropy parameter is γ = 1.28. It is suggestive that the flux pinning of the film can be attributed to the mixed randomly distributed point defects and mass anisotropy. The *f*_p_-*h* curves of the (Li,Fe)OHFeSe film before and after Mn doping are both close to the surface pinning [58]. This intrinsic pinning is contributed by the insulating (Li,Fe)OH spacer layer.

**Figure 14:**
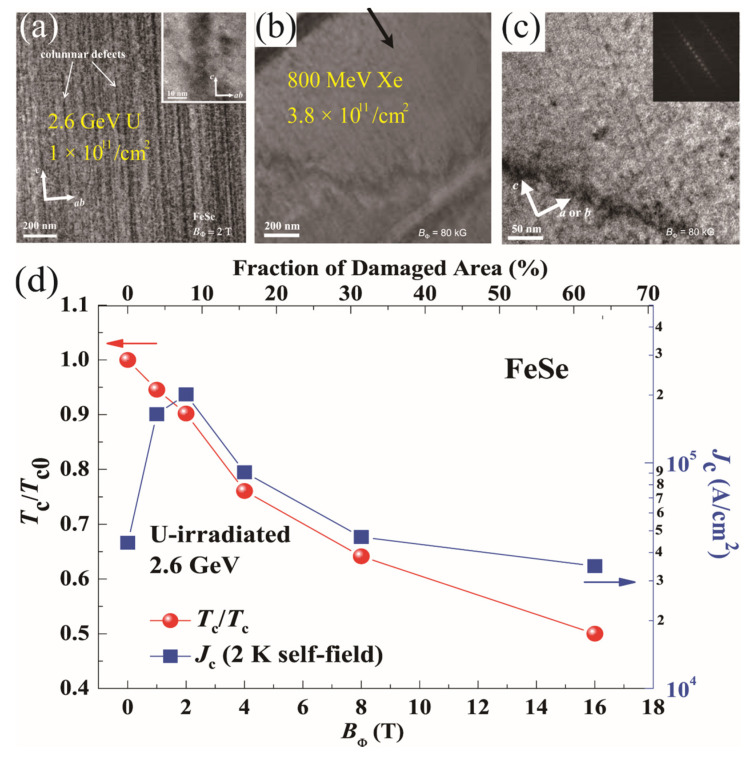
(**a**) Cross-sectional TEM micrograph of FeSe irradiated by uranium with *B_φ_* = 2 T. The inset shows an enlarged view of the columnar defect [61]. (**b**,**c**) TEM images of the FeTe_0.61_Se_0.39_ single crystals irradiated by 800 MeV Xe at B*_φ_* = 8 T. The inset in (**c**) is the Fourier transformation [62]. (**d**) Normalized T_c_ (T_c_/T_c0_, where T_c0_ is for the pristine one) and the self-field *J*_c_ at 2 K as a function of the matching field *B_φ_* (bottom axis) and damaged area (top axis) for the uranium-irradiated FeSe.

The naturally formed pinning centers are quite rare in iron–chalcogenide superconductors, making it necessary to introduce artificial pinning centers. Ion irradiation is considered to be an effective method for introducing point and column defects depending on the type and energy of the irradiation particles [72]. So far, several groups have performed irradiation experiments on the Fe(Te,Se) single crystals and films. As summarized in Figure 14, the large-sized particles, such as U, Xe, and Au ions, created continuous columnar defects in the FeSe (Figure 14a) and FeTe_0.61_Se_0.39_ (Figure 14b,c) single crystals. As shown in Figure 14d, the U-irradiation suppressed the T_c_ of FeSe even at a small dose, which demonstrates a nearly linear dependence of T_c_ on the dose [61]. The quick suppression of T_c_ when compared with iron pnictides can be explained by the much larger damaged areas (~10 nm) shown in the inset of Figure 14a. In the Ba_0.6_K_0.4_Fe_2_As_2_ single crystals, the damaged area is only ~2–5 nm. Consequently, one can observe a peak of the *J*_c_-B_Φ_ curve at a small B_Φ_ =2 T, above which the *J*_c_ is quickly lowered due to the damaged superconducting matrix. 

**Figure 15:**
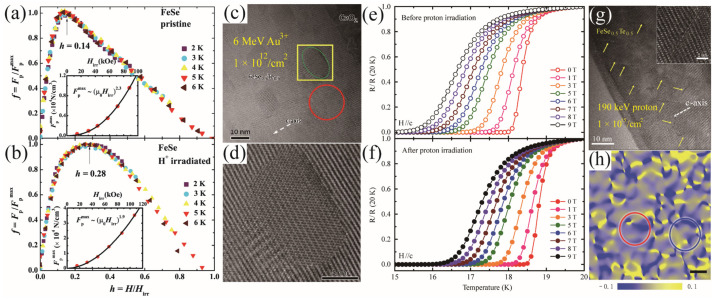
(**a**,**b**) show the normalized flux pinning force *f* = *F*_p_/*F*_p,max_ as a function of reduced field *h* = *H*/*H*_max_ of the FeSe single crystals before and after irradiation [75]. (**c**,**d**) High-resolution TEM image of Au-ion-irradiated FeSe_0.5_Te_0.5_ film [73]. (**e**,**f**) Temperature dependence of normalized resistivity of the FeSe_0.5_Te_0.5_ (FST) film before and after irradiation by the 190 keV protons [74]. (**g**) HRTEM image of FST film irradiated with protons. (**h**) In-plane strain map (*ε*_zz_). The color bar in the middle indicates the strain from -0.1 (compressive) to 0.1 (tensile).

Ozaki et al. studied the irradiation effect in increasing *J*_c_. For the low-energy Au irradiation, it produces cluster-like defects with sizes of 10–15 nm (Figure 15c,d), much larger than the coherence length [73]. The strong isotropic pinning leads to a nearly 70% increase of *J*_c_ at 9 T and 10 K with H//c. The low-energy proton irradiation introduces splayed cascade defects, which are about 1–2 nm in diameter and ~10 nm wide, in the FeTe_1−x_Se_x_ films deposited on the CeO_2_ buffer layer, as shown in Figure 15g. The cascade defects cause strained areas [74]. The blue valleys are the highly compressed regions, whereas the yellow peak regions indicate tensile strain (Figure 15h). The nanoscale strain and proximity effect causes an enhanced T_c_ from 18 K to 18.5 K, as well as the increased H_irr_, H_c2_, and *J*_c_ (Figure 15e,f). A similar proton irradiation effect can be found in the FeSe single crystals. Figure 15a,b show that the *h*_peak_ moves from 0.14 to 0.28, indicating the introduction of point defects [75]. The self-field *J*_c_ of the irradiated FeSe single crystals was enhanced from 3 × 10^4^ A/cm^2^ to 8 × 10^4^ A/cm^2^.

## 5. Practical Applications of Iron–Chalcogenide Superconductors

### 5.1. Fabrications of FeSe Film for Superconducting Radio-Frequency Applications

**Figure 16:**
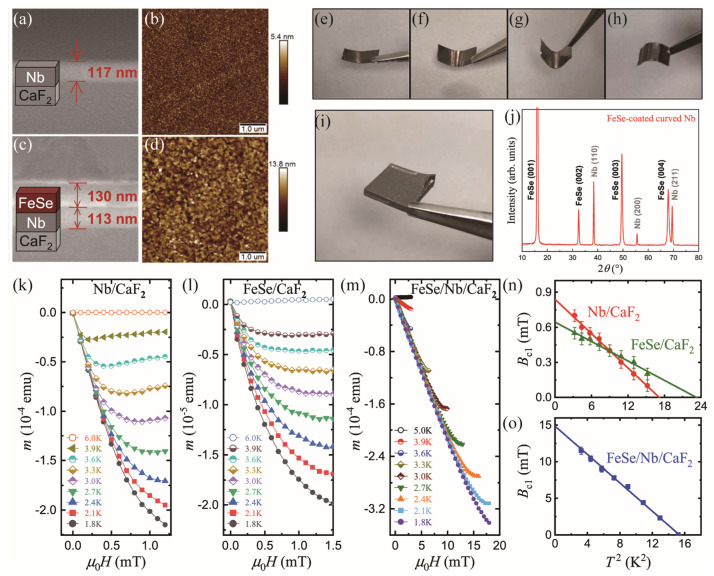
SEM cross-sectional images of (**a**) Nb film grown on CaF_2_ and (**b**) FeSe film on Nb-coated CaF_2_ films [76]. (**c**,**d**) Atomic force microscopy (AFM) images (5 × 5 µm^2^) of Nb/CaF_2_ and FeSe/Nb/CaF_2_ films. (**e**–**h**) The FeSe-coated Nb foil bent to inward angles of 160°, 120°, and 60° and an outward angle of 90°. (**i**,**j**) Photo and *θ*–2*θ* scans of FeSe film deposited on a curved bulk Nb. (**k**–**m**) *m*(*H*) curves of Nb/CaF_2_, FeSe/CaF_2_, and FeSe/Nb/CaF_2_ at various temperatures for H parallel to the film surface. (**n**,**o**) *T*^2^ dependence of *B*_c1_ for Nb/CaF_2_, FeSe/CaF_2_, and FeSe/Nb/CaF_2_.

Bulk Nb superconducting radio-frequency (SRF) cavities have been widely utilized in accelerators. However, the gradient of acceleration and overall performance are greatly limited by the overheating field (*B*_sh_). Lin et al. fabricated the FeSe-coated Nb as an alternative material. The out-of-plane *θ–2θ* XRD shows only the (00l) peaks of the FeSe film, indicating excellent *c*-axis orientation and crystallinity. As shown in Figure 16a–d, SEM and AFM images demonstrate a homogeneous thickness distribution of Nb (117 nm) and FeSe (130 nm) layers throughout the film, with root mean square roughness of 0.773 nm for Nb/CaF_2_ and 1.89 nm for FeSe/Nb/CaF_2_. This is beneficial to the effectiveness of the Bean–Livingston barrier, which can prevent vortex penetration and increase the *B*_sh_.

As shown in Figure 16k–m, the *m*(*H*) curves of the Nb/CaF_2_, FeSe/CaF_2_, and FeSe/Nb/CaF_2_ film deviate at different temperatures and fields. The *B*_c1_(*T*) is obtained based on the deviation point from the linear background (Figure 16n,o). After the deposition of FeSe on Nb/CaF_2_, the *B*_c1_(0) is over ten times higher than that of Nb/CaF_2_ and FeSe/CaF_2_. In addition, the FeSe layer exhibits excellent bending ability. Figure 16e–h indicate that the FeSe layer maintained a strong adhesion to the surface, even when subjected to varying degrees of inward or outward bending, demonstrating extraordinary resilience. Furthermore, FeSe film can be fabricated on a 10 × 10 mm^2^ curved Nb cut from a cavity, as shown in Figure 16i. The XRD pattern exhibits the (00l) crystal orientation on the polycrystalline Nb substrate (Figure 16j). It suggests that FeSe satisfies the basic requirements and can be an alternative material for SRF cavities.

### 5.2. Fabrications of Meters-Long FeTe_1−x_Se_x_-Coated Conductors and the Applications in Coils

**Figure 17:**
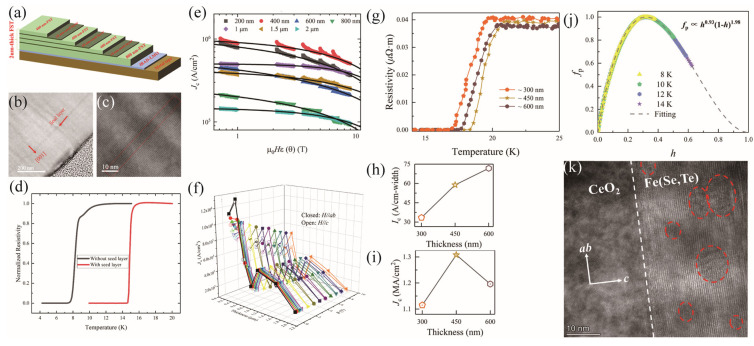
Fabrications of thick Fe(Se,Te) (FST) film. (**a**) The structure of a 2 μm thick FST film [65]. (**b**) Cross-sectional bright-field TEM image of 1 μm thick FST films. The arrow indicates the seed layer grown at high temperatures. (**c**) An enlarged image of the seed layer region in panel (**b**). (**d**) Normalized resistivity near T_c_ for the thick film with and without seed layer. (**e**) Scaling behavior of *J*_c_(*θ*) at 4.2 K for the films with different thicknesses. (**f**) *J*_c_(4.2 K) vs. film thickness under different magnetic fields with *H*//*ab* and *H* //*c*. (**g**) *ρ*-T curve, (**h**) self-field critical current, and (**i**) critical current density at 4.2 K of FST films with different thicknesses [74]. (**j**) Normalized pinning force densities of 450 nm thick FST films at different temperatures with a field parallel to the *c*-axis. (**k**) Cross-sectional TEM image of the 450 nm thick FST film.

Like other siblings of iron-based superconductors, the 11 system exhibits a weak link effect. The critical current density across the junction of the FeSe_0.5_Te_0.5_ films on the [001]-tilt SrTiO_3_ (STO) bicrystals decreases exponentially when the misorientation angle is larger than a critical value of *θ*_c_ = 9° [77,78,79]. As a result, the epitaxial film is an accessible option to reach a large supercurrent. The initiative efforts were mainly devoted to growing films on single crystalline substrates. Bellingeri et al. tried growing FeTe_1−x_Se_x_ thin film on (001)-orientated MgO, STO, LaAlO_3_ (LAO), and yttria-stabilized zirconia (ZrO:Y) using the PLD method. They found that the T_c_ is independent on the film thickness but closely correlated to the compressive strain [80]. The highest T_c_ = 21 K was obtained in the film deposited on the LAO substrates. The CaF_2_ substrate was also used, and a 1 MA/cm^2^ critical current density was achieved at 4.2 K and 0 T [70]. The 5–20 nm lattice disorder contributes to the complete isotropy of the current carrying ability. Nowadays, significant progress has been made in research on Fe(Se,Te)-coated conductors (CC). Si et al. significantly enhanced the superconducting performance of Fe(Se,Te) CC by employing a CeO_2_ buffer layer [74]. The FeSe_0.5_Te_0.5_ on a rolling-assisted biaxially textured substrate (RABiTS) exhibited T_c_ > 20 K. The self-field *J_c_* reached 1 MA/cm^2^ at 4.2 K, and *J_c_* remained around 1 × 10^5^ A/cm^2^ under a high magnetic field of 30 T. Sylva et al. found that the CeO_2_ layer plays an important role in preventing the element diffusion from the Ni-W substrate and inducing in-plane texture [81,82]. Song et al. adopted an interface engineering strategy to mitigate the thickness effect, successfully fabricating micron-thick FeSe_0.5_Te_0.5_ superconducting films on IBAD-LaMnO_3_-buffered metal tapes [65]. Unlike the continuous growth mode, the alternating growth of a 10 nm thick seed layer and a 400 nm thick FST superconducting layer ensures the crystalline quality of FeSe_0.5_Te_0.5_ films (see Figure 17a–c). Apart from the minor pits and droplets, the elemental distribution of the film is uniform, with a smooth surface morphology. Figure 17d depicts the normalized resistance curves of FST films under zero magnetic field, revealing superconductivity across all films. As the film thickness increases, the influence of the LMO buffer layer on T_c_ diminishes, and the introduction of the seed layer promotes the uniform growth of epitaxy film. Consequently, T_c_ gradually increases and approaches the bulk value of ~14–15 K. In Figure 17f, the highest *J*_c_ appears in the 400 nm thick film, with a self-field *J*_c_ of ∼1.3 MA/cm^2^ and *J*_c_(9 T) of 0.71 and 0.5 MA/cm^2^ for H//*ab* and H//*c*, respectively. Upon further increasing the thickness, there is a rapid drop down of *J*_c_, suggesting the presence of the thickness effect. But, with the help of the seed layer, the *J*_c_ revives to a second peak at 1 μm. A possible reason for the thickness effect is the sensitivity of pinning effects to temperature and thickness in type-II superconductors, where *δl* pinning contributes more to *J_c_* than *δ*T_c_ pinning. When the film thickness is 200 nm, *δl* pinning dominates. With increasing thickness and temperature, *δl* pinning gradually transforms to *δ*T_c_ pinning and exhibits a mixed form.

Ye et al. investigated the influence of substrate temperature and thickness on the superconducting properties of FeSe_0.5_Te_0.5_ CC. Moderate substrate temperatures are conducive to achieving superconducting films with high performance due to the impact on the evaporation of Se and Te, leading to improved texture and stoichiometry. Based on this, increasing the thickness leads to a transition in the deposition mode from heteroepitaxial to homoepitaxial growth, and the prolonged deposition mitigates longitudinal elemental non-uniformity and the attraction of metal ions within the buffer layer, enabling the attainment of good texture even at low temperatures. However, as the thickness increases, the phenomenon of compositional inhomogeneity in the film becomes more severe. Additionally, the actual temperature at the deposition surface may be lower than the set temperature, leading to grain misorientation or the formation of non-superconducting phases, which is detrimental to the superconducting performance of Fe(Se,Te) CC. As shown in Figure 17g–i, the T_c_ first increases at 450 nm and then decreases for a little bit at 600 nm. The corresponding *J*_c_ peaks at 450 nm, but the *I*_c_ continues to grow larger with the increasing thickness. The analysis of the pinning mechanism revealed the dominance of point pinning, as proved by the *h*_peak_~0.33 in Figure 17j and 3–10 nm defects in the TEM image in Figure 17k, which do not change with the temperature.

**Figure 18:**
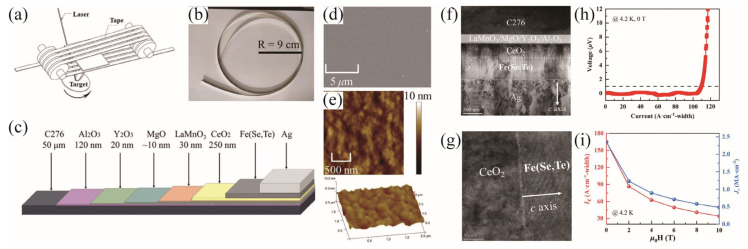
(**a**) Sketch of reel-to-reel PLD [83]. (**b**) Photo image of 1 m long Fe(Se,Te) CC. (**c**) Structure diagram, (**d**) SEM image, and (**e**) AFM image of the Fe(Se,Te) CC. (**f**) Low- and (**g**) high-magnification cross-sectional TEM images of the Fe(Se,Te) CC. (**h**) End-to-end *I*_c_ at 4.2 K and self-field of 1 m long Fe(Se,Te) CC. (**i**) *I*_c_ and *J*_c_ as a function of magnetic fields up to 10 T with H//c at 4.2 K for short sample.

Liu et al. creatively utilized reel-to-reel PLD for the first time to fabricate 1 m long Fe(Se,Te) coated conductors with high superconducting performance [83], as shown in Figure 18a. Figure 18d demonstrates the smooth surface of the CC, except for some particles most likely formed by the condensation of Se vapor. The AFM image in Figure 18e also proves the smooth plane with a small square root mean square roughness of about 1 nm. Figure 18f,g illustrate the high-quality epitaxial growth of Fe(Se,Te) films on CeO_2_ buffer layers, where all (00l) planes are clearly visible with distinct interfaces between layers. Figure 18h,i demonstrate that the end-to-end *I*_c_ of the samples reached 108 A/cm at 4.2 K and self-field, with good performance observed even under high magnetic fields for short samples. Sharp superconducting transitions occurred in Fe(Se,Te) films under magnetic fields ranging from 0 to 9 T, with T_c_ reaching up to 17.5 K, and an estimated *H*_c2_(0) = 53 T. The *J*_c_(4.2 K) derived from the Bean model is over 2 MA/cm^2^ at self-field.

**Figure 19:**
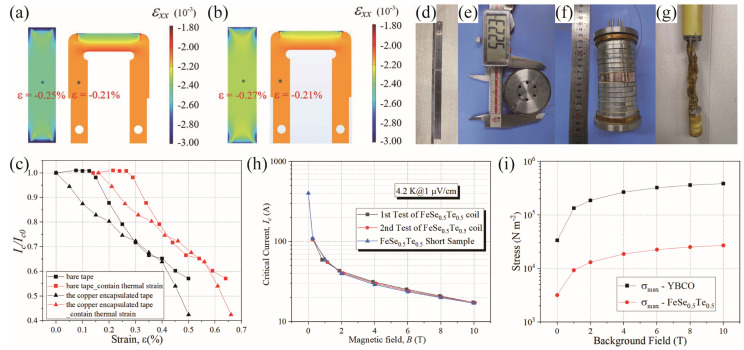
(**a**,**b**) The finite element method (FEM) calculations of the strain of the bare FeSe_0.5_Te_0.5_ tape and the copper-encapsulated FeSe_0.5_Te_0.5_ tape during the cooling process from 343 K to 4.2 K [84]. (**c**) The normalized critical current *I_c_*/*I_c0_* of the bare and the copper-encapsulated FeSe_0.5_Te_0.5_ tapes after considering the influence of the cooling process. (**d**) The outer view of FeSe_0.5_Te_0.5_-encapsulated tape. (**e**) The FeSe_0.5_Te_0.5_ single pancake coils (SPC). (**f**) The insert hybrid magnet. (**g**) Part of the testing device [85]. (**h**) Magnetic field dependence of transport *I_c_* for FeSe_0.5_Te_0.5_ insert coil in the first and second tests. (**i**) Maximum stress on the FeSe_0.5_Te_0.5_ coil and the YBCO coils at the *I*_c_ of FeSe_0.5_Te_0.5_ tape.

Liu et al. optimized the ability of the Fe(Se,Te)-coated conductors to withstand uniaxial tensile pressure by encapsulating them with a copper layer [84]. As illustrated in Figure 19a–c, regardless of considering the cooling process effects, the mechanical and electrical performance of the tape with copper encapsulation under tensile strain is consistently superior to that of the bare tape. As the strain gradually increased from 0% to 0.5%, the *I*_c_ of both the bare tape and the copper-coated tape decreased by approximately 58% and 43%, respectively. With the adoption of copper encapsulation, the irreversible tensile strain limit of the FeSe_0.5_Te_0.5_ tape reached 0.15%. Furthermore, considering the effects of the cooling process, the actual irreversible tensile strain limit was enhanced to 0.29%.

Wei et al. successfully fabricated the world’s first hybrid coil by using FeSe_0.5_Te_0.5_ CC. The schematic diagram of the FeSe_0.5_Te_0.5_ hybrid coil and the inserted magnet is shown in Figure 19d–g. This hybrid coil consists of a single-layer FeSe_0.5_Te_0.5_ coil with an inner diameter of 51 mm and eight double-layer YBCO coils with inner diameters of 45 mm. The YBCO coils are divided into two parts and connected to both sides of the FeSe_0.5_Te_0.5_ coil. Additionally, a copper layer is used to cover the FeSe_0.5_Te_0.5_ coil to withstand the high stress during cooling, thereby preventing substrate shrinkage from damaging the superconducting layer. Figure 19i shows that the maximum stress on the coil is less than 1 MPa up to 10 T, which is within the allowable pressure range of the tape. As shown in Figure 19h, based on the quench process of the FeSe_0.5_Te_0.5_ coil in the first cold test, the self-field *I*_c_ reached 108.1 A, and the *I*_c_ at a 10T field reached 17.4 A. The *J_c_* was approximately 1.42 × 10^5^ A/cm^2^, which is consistent with the results of the short sample test. Results of repeated cold tests demonstrate the stable performance of the FeSe_0.5_Te_0.5_ hybrid coil.

## 6. Conclusions and Prospects

Despite the electroneutral FeSe layer, iron–chalcogenide superconductors present varieties of crystal structures exhibiting different superconducting characteristics. The interesting properties of this system prompt scientists to invent different methodologies to modulate the crystalline and electrical structures through oxidation, intercalation, the electrochemical method, and interface engineering, which greatly enriche the fabrications and investigations of high-temperature superconductors. According to the summarized phase diagram, higher T_c_ could be achieved through charge transfer and electron–phonon coupling across heterostructures. More importantly, the FeSe_1−x_Te_x_ and the intercalated FeSe have a large upper critical field, which is the precondition for high-field applications. The successful fabrications of meters-long FeSe_1−x_Te_x_ CC facilitate the first fabrications of insert pancake coils. Compared with the poor texture in the iron-based superconducting wires fabricated through the PIT process, the in-plane texture can be easily obtained in the CC, leading to a higher *J*_c_ than the FeSe_1−x_Te_x_ wires at high fields [86,87,88]. Due to the larger critical misorientation angle compared to cuprates (*θ*_c_ = 5°), less textured substrates are required for the FeSe_1−*x*_Te*_x_* CC. However, the vortex pinning is dominated by the scarce point defects, whether in single crystals or CC. Stronger vortex pinning centers, e.g., nanoparticles and correlated column defects introduced in the ReBa_2_Cu_3_O_7−*δ*_ (Re = rare earth elements) CC by mature techniques, are highly desirable for further enhancement of the current carrying ability of the iron chalcogenide superconductors.

## Figures and Tables

**Table 1:** Penetration depth, coherence length, and critical current density *J*_c_^ab^(0 T) of practical iron–chalcogenide superconductors. The anisotropy parameter is derived from Figure 11b. The pristine and irradiated samples are denoted as *pri* and *irr*, respectively. The flux pinning efficiency is calculated by *η* = *J*_c_*/J*_d_ at 0 T.

Materials	FeSe		FeTe_1−x_Se_x_		(Li,Fe)OHFeSe
**λ_ab_(0 K), nm**	445 [55,56]		430		281 [57,58]
**ξ_ab_(0 K), nm**	4.4 [56,59]		1.5 [60]		2.21 [50,58]
***J*_d_(0 K), MA/cm^2^**	11 [56]		36		57.7 [58]
**γ = B_c2_^ab^/B_c2_^c^**	2–3		1–3		2–5
***J*_c_-single crystal, MA/cm^2^**	0.043 (2 K, *pri*)	0.2 (2 K, *irr*) [61]	0.27 (2 K, *pri*)	0.52 (2 K, *irr*) [62]	0.24 [63]
***J*_c_-film, MA/cm^2^**	1.7 (2 K, Mono-layer) [34]		1.36 (4.2 K)		\
***J*_c_ coated conductor @ 4.2 K, MA/cm^2^**	\		0.43 [64]	1.3 [65]	\
***Maximum η***	15%		3.7%		0.4%

## Data Availability

Not applicable.

## References

[1] Kamihara Y., Watanabe T., Hirano M., Hosono H. (2008). Iron-Based Layered Superconductor La[O_1−*x*_F*_x_*]FeAs (*x* = 0.05−0.12) with T_c_ = 26 K. J. Am. Chem. Soc..

[2] Mazin I.I. (2010). Superconductivity gets an iron boost. Nature.

[3] Putti M., Pallecchi I., Bellingeri E., Cimberle M.R., Tropeano M., Ferdeghini C., Palenzona A., Tarantini C., Yamamoto A., Jiang J. (2010). New Fe-based superconductors: Properties relevant for applications. Supercond. Sci. Technol..

[4] Yao C., Ma Y. (2019). Recent breakthrough development in iron-based superconducting wires for practical applications. Supercond. Sci. Technol..

[5] Takahashi H., Igawa K., Arii K., Kamihara Y., Hirano M., Hosono H. (2008). Superconductivity at 43 K in an iron-based layered compound LaO_1−*x*_F*_x_*FeAs. Nature.

[6] Hsu F.-C., Luo J.-Y., Yeh K.-W., Chen T.-K., Huang T.-W., Wu P.M., Lee Y.-C., Huang Y.-L., Chu Y.-Y., Yan D.-C. (2008). Superconductivity in the PbO-type structure α-FeSe. Proc. Natl. Acad. Sci. USA.

[7] Pelliciari J., Karakuzu S., Song Q., Arpaia R., Nag A., Rossi M., Li J., Yu T., Chen X., Peng R. (2021). Evolution of spin excitations from bulk to monolayer FeSe. Nat. Commun..

[8] Bao W., Qiu Y., Huang Q., Green M.A., Zajdel P., Fitzsimmons M.R., Zhernenkov M., Chang S., Fang M., Qian B. (2009). Tunable (δπ, δπ)-Type Antiferromagnetic Order in α-Fe(Te,Se) Superconductors. Phys. Rev. Lett..

[9] Fang M., Wang H., Dong C., Li Z., Feng C., Chen J., Yuan H. (2011). Fe-based superconductivity with Tc = 31 K bordering an antiferromagnetic insulator in (Tl,K)Fe_x_Se_2_. Europhys. Lett..

[10] Bao W., Huang Q.-Z., Chen G.-F., Wang D.-M., He J.-B., Qiu Y.-M. (2011). A Novel Large Moment Antiferromagnetic Order in K_0.8_Fe_1.6_Se_2_ Superconductor. Chin. Phys. Lett..

[11] Burrard-Lucas M., Free D.G., Sedlmaier S.J., Wright J.D., Cassidy S.J., Hara Y., Corkett A.J., Lancaster T., Baker P.J., Blundell S.J. (2013). Enhancement of the superconducting transition temperature of FeSe by intercalation of a molecular spacer layer. Nat. Mater..

[12] Shi M.Z., Wang N.Z., Lei B., Shang C., Meng F.B., Ma L.K., Zhang F.X., Kuang D.Z., Chen X.H. (2018). Organic-ion-intercalated FeSe-based superconductors. Phys. Rev. Mater..

[13] Lu X.F., Wang N.Z., Wu H., Wu Y.P., Zhao D., Zeng X.Z., Luo X.G., Wu T., Bao W., Zhang G.H. (2014). Coexistence of superconductivity and antiferromagnetism in (Li_0.8_Fe_0.2_)OHFeSe. Nat. Mater..

[14] Fang M.H., Pham H.M., Qian B., Liu T.J., Vehstedt E.K., Liu Y., Spinu L., Mao Z.Q. (2008). Superconductivity close to magnetic instability in Fe(Se_1−*x*_Te*_x_*)_0.82_. Phys. Rev. B.

[15] Dong C., Wang H., Yang J., Qian B., Chen J., Li Z., Yuan H., Fang M. (2010). Effect of annealing on superconductivity in Fe_1+*y*_(Te_1−*x*_S*_x_*) system. Sci. China Phys. Mech. Astron..

[16] Sun Y., Tsuchiya Y., Taen T., Yamada T., Pyon S., Sugimoto A., Ekino T., Shi Z., Tamegai T. (2014). Dynamics and mechanism of oxygen annealing in Fe_1+y_Te_0.6_Se_0.4_ single crystal. Sci. Rep..

[17] Liu T.J., Ke X., Qian B., Hu J., Fobes D., Vehstedt E.K., Pham H., Yang J.H., Fang M.H., Spinu L. (2009). Charge-carrier localization induced by excess Fe in the superconductor Fe_1+*y*_Te_1−*x*_Se*_x_*. Phys. Rev. B.

[18] Sun Y., Shi Z., Tamegai T. (2019). Review of annealing effects and superconductivity in Fe_1+y_Te_1−x_Se_x_ superconductors. Supercond. Sci. Technol..

[19] Dong C., Wang H., Li Z., Chen J., Yuan H.Q., Fang M. (2011). Revised phase diagram for the FeTe_1−*x*_Se*_x_* system with fewer excess Fe atoms. Phys. Rev. B.

[20] Ding X., Fang D., Wang Z., Yang H., Liu J., Deng Q., Ma G., Meng C., Hu Y., Wen H.-H. (2013). Influence of microstructure on superconductivity in K_x_Fe_2−y_Se_2_ and evidence for a new parent phase K_2_Fe_7_Se_8_. Nat. Commun..

[21] Li W., Ding H., Deng P., Chang K., Song C., He K., Wang L., Ma X., Hu J.-P., Chen X. (2012). Phase separation and magnetic order in K-doped iron selenide superconductor. Nat. Phys.

[22] Ying T.P., Chen X.L., Wang G., Jin S.F., Zhou T.T., Lai X.F., Zhang H., Wang W.Y. (2012). Observation of superconductivity at 30∼46K in A_x_Fe_2_Se_2_ (A = Li, Na, Ba, Sr, Ca, Yb and Eu). Sci. Rep..

[23] Ying T., Chen X., Wang G., Jin S., Lai X., Zhou T., Zhang H., Shen S., Wang W. (2013). Superconducting Phases in Potassium-Intercalated Iron Selenides. J. Am. Chem. Soc..

[24] Guo J., Lei H., Hayashi F., Hosono H. (2014). Superconductivity and phase instability of NH_3_-free Na-intercalated FeSe_1−z_S_z_. Nat. Commun..

[25] Sun S., Wang S., Yu R., Lei H. (2017). Extreme anisotropy and anomalous transport properties of heavily electron doped Li_x_(NH_3_)_y_Fe_2_Se_2_ single crystals. Phys. Rev. B.

[26] Hatakeda T., Noji T., Sato K., Kawamata T., Kato M., Koike Y. (2016). New Alkali-Metal- and 2-Phenethylamine-Intercalated Superconductors *A_x_*(C_8_H_11_N)*_y_*Fe_1−*z*_Se(*A* = Li, Na) with the Largest Interlayer Spacings and *T*_c_∼40 K. J. Phys. Soc. Jpn..

[27] Meng Y., Xing X., Yi X., Li B., Zhou N., Li M., Zhang Y., Wei W., Feng J., Terashima K. (2022). Protonation-induced discrete superconducting phases in bulk FeSe single crystals. Phys. Rev. B.

[28] Lei B., Cui J.H., Xiang Z.J., Shang C., Wang N.Z., Ye G.J., Luo X.G., Wu T., Sun Z., Chen X.H. (2016). Evolution of High-Temperature Superconductivity from a Low-Tc Phase Tuned by Carrier Concentration in FeSe Thin Flakes. Phys. Rev. Lett..

[29] Ying T.P., Wang M.X., Wu X.X., Zhao Z.Y., Zhang Z.Z., Song B.Q., Li Y.C., Lei B., Li Q., Yu Y. (2018). Discrete Superconducting Phases in FeSe-Derived Superconductors. Phys. Rev. Lett..

[30] Lu X.F., Wang N.Z., Zhang G.H., Luo X.G., Ma Z.M., Lei B., Huang F.Q., Chen X.H. (2014). Superconductivity in LiFeO_2_Fe_2_Se_2_ with anti-PbO-type spacer layers. Phys. Rev. B.

[31] Sun H., Woodruff D.N., Cassidy S.J., Allcroft G.M., Sedlmaier S.J., Thompson A.L., Bingham P.A., Forder S.D., Cartenet S., Mary N. (2015). Soft Chemical Control of Superconductivity in Lithium Iron Selenide Hydroxides Li_1–*x*_Fe*_x_*(OH)Fe_1–*y*_Se. Inorg. Chem..

[32] Dong X., Jin K., Yuan D., Zhou H., Yuan J., Huang Y., Hua W., Sun J., Zheng P., Hu W. (2015). (Li_0.84_Fe_0.16_)OHFe_0.98_Se superconductor: Ion-exchange synthesis of large single-crystal and highly two-dimensional electron properties. Phys. Rev. B.

[33] Wang Q.-Y., Li Z., Zhang W.-H., Zhang Z.-C., Zhang J.-S., Li W., Ding H., Ou Y.-B., Deng P., Chang K. (2012). Interface-Induced High-Temperature Superconductivity in Single Unit-Cell FeSe Films on SrTiO_3_. Chin. Phys. Lett..

[34] Zhang W.-H., Sun Y., Zhang J.-S., Li F.-S., Guo M.-H., Zhao Y.-F., Zhang H.-M., Peng J.-P., Xing Y., Wang H.-C. (2014). Direct Observation of High-Temperature Superconductivity in One-Unit-Cell FeSe Films. Chin. Phys. Lett..

[35] Zhang H., Zhang D., Lu X., Liu C., Zhou G., Ma X., Wang L., Jiang P., Xue Q.-K., Bao X. (2017). Origin of charge transfer and enhanced electron–phonon coupling in single unit-cell FeSe films on SrTiO_3_. Nat. Commun..

[36] Ge J.-F., Liu Z.-L., Liu C., Gao C.-L., Qian D., Xue Q.-K., Liu Y., Jia J.-F. (2015). Superconductivity above 100 K in single-layer FeSe films on doped SrTiO_3_. Nat. Mater..

[37] Hou Q., Sun L., Sun Y., Shi Z. (2023). Review of Single Crystal Synthesis of 11 Iron-Based Superconductors. Materials.

[38] Yi X., Xing X., Qin L., Feng J., Li M., Zhang Y., Meng Y., Zhou N., Sun Y., Shi Z. (2021). Hydrothermal synthesis and complete phase diagram of FeSe_1−*x*_S*_x_* (0 ≤ *x* ≤ 1) single crystals. Phys. Rev. B.

[39] Terao K., Kashiwagi T., Shizu T., Klemm R.A., Kadowaki K. (2019). Superconducting and tetragonal-to-orthorhombic transitions in single crystals of FeSe_1−*x*_Te*_x_* (0 ≤ *x* ≤ 0.61 ). Phys. Rev. B.

[40] Sun Q., Xu Z., Dong C., Huang H., Wang D., Zhang X., Ma Y. (2023). Macroscopically ordered phase separation: A new strategy for improving the superconducting performance in Fe(Se, Te). Mater. Today Adv..

[41] Zhuang J., Yeoh W.K., Cui X., Xu X., Du Y., Shi Z., Ringer S.P., Wang X., Dou S.X. (2014). Unabridged phase diagram for single-phased FeSe_x_Te_1−x_ thin films. Sci. Rep..

[42] Fan F., Xu Z., Cheng Z., Huang H., Zhu Y., Liu S., Dong C., Zhang X., Ma Y. (2019). Enhanced superconducting properties of FeSe_0.8_Te_0.2_ thin films grown by pulsed laser deposition. Phys. C Supercond. Its Appl..

[43] Zhang H.-F., Chen X.-H., Xiao Q.-L., Chen F., Feng Z., Cao S., Zhang J., Qi Y., Shi Z., Ge J.-Y. (2021). Evolution of Superconducting Properties in Fe_1.1_Se_0.8_Te_0.2_ Films Before and After Structure Avalanche. ACS Appl. Mater. Interfaces.

[44] Dong C., Wang H., Mao Q., Khan R., Zhou X., Li C., Yang J., Chen B., Fang M. (2013). Phase diagram and annealing effect for Fe_1+δ_Te_1-x_S_x_ single crystals. J. Phys. Condens. Matter.

[45] Zhao C., Yi X., Hou Q., Feng J., Zhang Y., Xu M., Shi Z. (2021). Hydrothermal Synthesis and Transport Properties of FeS_1−*x*_Te*_x_* (0 ≤ *x* ≤ 0.15) Single Crystals. J. Supercond. Nov. Magn..

[46] Lai X., Zhang H., Wang Y., Wang X., Zhang X., Lin J., Huang F. (2015). Observation of Superconductivity in Tetragonal FeS. J. Am. Chem. Soc..

[47] Rebec S.N., Jia T., Zhang C., Hashimoto M., Lu D.-H., Moore R.G., Shen Z.-X. (2017). Coexistence of Replica Bands and Superconductivity in FeSe Monolayer Films. Phys. Rev. Lett..

[48] Miyata Y., Nakayama K., Sugawara K., Sato T., Takahashi T. (2015). High-temperature superconductivity in potassium-coated multilayer FeSe thin films. Nat. Mater..

[49] Farrar L.S., Bristow M., Haghighirad A.A., McCollam A., Bending S.J., Coldea A.I. (2020). Suppression of superconductivity and enhanced critical field anisotropy in thin flakes of FeSe. npj Quantum Mater..

[50] Wang Z., Yuan J., Wosnitza J., Zhou H., Huang Y., Jin K., Zhou F., Dong X., Zhao Z. (2017). The upper critical field and its anisotropy in (Li _1−*x*_Fe*_x_* )OHFe_1−*y*_Se. J. Phys. Condens. Matter.

[51] Pan Y., Sun Y., Zhou N., Yi X., Hou Q., Wang J., Zhu Z., Mitamura H., Tokunaga M., Shi Z. (2024). Novel anisotropy of upper critical fields in Fe_1+y_Te_0.6_Se_0.4_. J. Alloys Compd..

[52] Lei H., Petrovic C. (2011). Giant increase in critical current density of K_x_Fe_2−y_Se_2_ single crystals. Phys. Rev. B.

[53] Li M., Chen L., You W.-L., Ge J., Zhang J. (2014). Giant increase of critical current density and vortex pinning in Mn doped K_x_Fe_2−y_Se_2_ single crystals. Appl. Phys. Lett..

[54] Lei H., Petrovic C. (2011). Critical current density and mechanism of vortex pinning in K_x_Fe_2−y_Se_2_ doped with S. Phys. Rev. B.

[55] Abdel-Hafiez M., Ge J., Vasiliev A.N., Chareev D.A., Van de Vondel J., Moshchalkov V.V., Silhanek A.V. (2013). Temperature dependence of lower critical field H_c1_(T) shows nodeless superconductivity in FeSe. Phys. Rev. B.

[56] Lanoël L., Haberkorn N., Nieva G. (2024). Flux creep regimes and vortex phase diagram in β-FeSe single crystals. Phys. C Supercond. Its Appl..

[57] Khasanov R., Zhou H., Amato A., Guguchia Z., Morenzoni E., Dong X., Zhang G., Zhao Z. (2016). Proximity-induced superconductivity within the insulating (Li_0.84_Fe_0.16_)OH layers in ( Li_0.84_Fe_0.16_)OHFe_0.98_Se. Phys. Rev. B.

[58] Li D., Yuan J., Shen P., Xi C., Tian J., Ni S., Zhang J., Wei Z., Hu W., Li Z. (2019). Giant enhancement of critical current density at high field in superconducting (Li,Fe)OHFeSe films by Mn doping. Supercond. Sci. Technol..

[59] Amigó M.L., Crivillero V.A., Franco D.G., Nieva G. (2014). Multiband character of *β*-FeSe: Angular dependence of the magnetoresistance and upper critical field. J. Phys. Conf. Ser..

[60] Klein T., Braithwaite D., Demuer A., Knafo W., Lapertot G., Marcenat C., Rodière P., Sheikin I., Strobel P., Sulpice A. (2010). Thermodynamic phase diagram of Fe(Se_0.5_Te_0.5_) single crystals in fields up to 28 tesla. Phys. Rev. B.

[61] Sun Y., Park A., Pyon S., Tamegai T., Kambara T., Ichinose A. (2017). Effects of heavy-ion irradiation on FeSe. Phys. Rev. B.

[62] Tamegai T., Taen T., Yagyuda H., Tsuchiya Y., Mohan S., Taniguchi T., Nakajima Y., Okayasu S., Sasase M., Kitamura H. (2012). Effects of particle irradiations on vortex states in iron-based superconductors. Supercond. Sci. Technol..

[63] Lin H., Xing J., Zhu X., Yang H., Wen H.-H. (2016). Robust superconductivity and transport properties in (Li_1−*x*_Fe*_x_*)OHFeSe single crystals. Sci. China Phys. Mech. Astron..

[64] Xu Z., Yuan P., Ma Y., Cai C. (2017). High-performance FeSe_0.5_Te_0.5_ thin films fabricated on less-well-textured flexible coated conductor templates. Supercond. Sci. Technol..

[65] Song J., Xu Z., Xiong X., Yuan W., Dong C., Sun Q., Tang M., Chen W., Tian H., Li J. (2023). Critical Role Played by Interface Engineering in Weakening Thickness Dependence of Superconducting and Structural Properties of FeSe_0.5_Te_0.5_-Coated Conductors. ACS Appl. Mater. Interfaces.

[66] Si W., Han S.J., Shi X., Ehrlich S.N., Jaroszynski J., Goyal A., Li Q. (2013). High current superconductivity in FeSe_0.5_Te_0.5_-coated conductors at 30 tesla. Nat. Commun..

[67] Yuan P., Xu Z., Ma Y., Sun Y., Tamegai T. (2016). Angular-dependent vortex pinning mechanism and magneto-optical characterizations of FeSe_0.5_Te_0.5_ thin films grown on CaF_2_ substrates. Supercond. Sci. Technol..

[68] Braccini V., Kawale S., Reich E., Bellingeri E., Pellegrino L., Sala A., Putti M., Higashikawa K., Kiss T., Holzapfel B. (2013). Highly effective and isotropic pinning in epitaxial Fe(Se,Te) thin films grown on CaF_2_ substrates. Appl. Phys. Lett..

[69] Yuan P., Xu Z., Zhang H., Wang D., Ma Y., Zhang M., Li J. (2015). High performance FeSe_0.5_Te_0.5_ thin films grown at low temperature by pulsed laser deposition. Supercond. Sci. Technol..

[70] Shahbazi M., Wang X.L., Dou S.X., Fang H., Lin C.T. (2013). The flux pinning mechanism, and electrical and magnetic anisotropy in Fe_1.04_Te_0.6_Se_0.4_ superconducting single crystal. J. Appl. Phys..

[71] Sang L., Maheswari P., Yu Z., Yun F.F., Zhang Y., Dou S., Cai C., Awana V.P.S., Wang X. (2017). Point defect induced giant enhancement of flux pinning in Co-doped FeSe_0.5_Te_0.5_ superconducting single crystals. AIP Adv..

[72] (2018). M Eisterer Radiation effects on iron-based superconductors. Supercond. Sci. Technol..

[73] Ozaki T., Wu L., Zhang C., Si W., Jie Q., Li Q. (2018). Enhanced critical current in superconducting FeSe_0.5_Te_0.5_ films at all magnetic field orientations by scalable gold ion irradiation. Supercond. Sci. Technol..

[74] Ozaki T., Wu L., Zhang C., Jaroszynski J., Si W., Zhou J., Zhu Y., Li Q. (2016). A route for a strong increase of critical current in nanostrained iron-based superconductors. Nat. Commun..

[75] Sun Y., Pyon S., Tamegai T., Kobayashi R., Watashige T., Shigeru Kasahara Y.M.T.S. (2015). Enhancement of critical current density and mechanism of vortex pinning in H_+_-irradiated FeSe single crystal. Appl. Phys. Express.

[76] Lin Z., Qin M., Li D., Shen P., Zhang L., Feng Z., Sha P., Miao J., Yuan J., Dong X. (2021). Enhancement of the lower critical field in FeSe-coated Nb structures for superconducting radio-frequency applications. Supercond. Sci. Technol..

[77] Si W., Zhang C., Shi X., Ozaki T., Jaroszynski J., Li Q. (2015). Grain boundary junctions of FeSe_0.5_Te_0.5_ thin films on SrTiO_3_ bi-crystal substrates. Appl. Phys. Lett..

[78] Sarnelli E., Nappi C., Camerlingo C., Enrico E., Bellingeri E., Kawale S., Braccini V., Leveratto A., Ferdeghini C. (2017). Properties of Fe(Se, Te) Bicrystal Grain Boundary Junctions, SQUIDs, and Nanostrips. IEEE Trans. Appl. Supercond..

[79] Iida K., Hänisch J., Yamamoto A. (2020). Grain boundary characteristics of Fe-based superconductors. Supercond. Sci. Technol..

[80] Bellingeri E., Pallecchi I., Buzio R., Gerbi A., Marrè D., Cimberle M.R., Tropeano M., Putti M., Palenzona A., Ferdeghini C. (2010). Tc = 21 K in epitaxial FeSe_0.5_Te_0.5_ thin films with biaxial compressive strain. Appl. Phys. Lett..

[81] Sylva G., Bellingeri E., Bernini C., Celentano G., Ferdeghini C., Leveratto A., Lisitskiy M., Malagoli A., Manca N., Mancini A. (2020). The role of texturing and thickness of oxide buffer layers in the superconducting properties of Fe(Se,Te) Coated Conductors. Supercond. Sci. Technol..

[82] Sylva G., Augieri A., Mancini A., Rufoloni A., Vannozzi A., Celentano G., Bellingeri E., Ferdeghini C., Putti M., Braccini V. (2019). Fe(Se,Te) coated conductors deposited on simple rolling-assisted biaxially textured substrate templates. Supercond. Sci. Technol..

[83] Liu L., Ye J., Mou S., Zhu R., Miao C., Li Y., Shi Z., Qi Y., Ge J., Liu H. (2023). Fabrication of Meter-Long Class Fe(Se,Te)-Coated Conductors with High Superconducting Performance. Adv. Eng. Mater..

[84] Liu X., Wei S., Shi Y., Liu F., Zhou C., Li Q., Li Y., Liu L., Shi Z., Ren L. (2022). Reversible critical current performance of FeSe_0.5_Te_0.5_ coated conductor tapes under uniaxial tensile strain. Supercond. Sci. Technol..

[85] Wei S., Liu X., Zhang Z., Liu F., Qin J., Shi Y., Li Y., Liu L., Shi Z., Ren L. (2023). First performance test of FeSe_0.5_Te_0.5_-coated conductor coil under high magnetic fields. Supercond. Sci. Technol..

[86] Zhang S., Feng J., Ma X., Liu J., Li C., Zhang P. (2017). In-situ and ex-situ PIT fabrication of FeSe superconducting tapes. J. Mater. Sci. Mater. Electron..

[87] Li X., Zhang Y., Yuan F., Zhuang J., Cao Z., Xing X., Zhou W., Shi Z. (2016). Fabrication of Nb-sheathed FeSe_0.5_Te_0.5_ tape by an ex-situ powder-in-tube method. J. Alloys Compd..

[88] Palombo M., Malagoli A., Pani M., Bernini C., Manfrinetti P., Palenzona A., Putti M. (2015). Exploring the feasibility of Fe(Se,Te) conductors by ex-situ powder-in-tube method. J. Appl. Phys..

